# Spatiotemporal analysis and forecasting of lumpy skin disease outbreaks in Ethiopia based on retrospective outbreak reports

**DOI:** 10.3389/fvets.2024.1277007

**Published:** 2024-03-12

**Authors:** Shimels Tesfaye, Fikru Regassa, Gashaw Beyene, Samson Leta, Jan Paeshuyse

**Affiliations:** ^1^Laboratory of Host–Pathogen Interaction, Department of Biosystems, Division of Animal and Human Health Engineering, KU Leuven, Leuven, Belgium; ^2^College of Veterinary Medicine and Agriculture, Addis Ababa University, Addis Ababa, Ethiopia; ^3^Ministry of Agriculture, Livestock and Fisheries, Addis Ababa, Ethiopia; ^4^Epidemiology Directorate, Ministry of Agriculture, Livestock and Fisheries, Addis Ababa, Ethiopia

**Keywords:** Ethiopia, forecast, lumpy skin disease, SaTScan, spatial, temporal, time series analysis

## Abstract

**Introduction:**

Lumpy skin disease is a viral disease that affects cattle belonging to genus Capripoxvirus (Poxviridae) and lead to significant economic losses.

**Objective:**

The objective of this study was to evaluate the distribution of lumpy skin disease (LSD) outbreaks and predict future patterns based on retrospective outbreak reports in Ethiopia.

**Methods:**

Data were collected through direct communication with regional laboratories and a hierarchical reporting system from the Peasant Associations to Ministry of Agriculture. Time-series data for the LSD outbreaks were analyzed using classical additive time-series decomposition and STL decomposition. Four models (ARIMA, SARIMA, ETS, STLF) were also used to forecast the number of LSD outbreaks that occurred each month for the years (2021–2025) after the models’ accuracy test was performed. Additionally, the space–time permutation model (STP) were also used to study retrospective space–time cluster analysis of LSD outbreaks in Ethiopia.

**Results:**

This study examined the geographical and temporal distribution of LSD outbreaks in Ethiopia from 2008 to 2020, reporting a total of 3,256 LSD outbreaks, 14,754 LSD-positive cases, 7,758 deaths, and 289 slaughters. It also covered approximately 68% of Ethiopia’s districts, with Oromia reporting the highest LSD outbreaks. In the LSD’s temporal distribution, the highest peak was reported following the rainy season in September to December and its lowest peak in the dry months of April and May. Out of the four models tested for forecasting, the SARIMA (3, 0, 0) (2, 1, 0) [12] model performed well for the validation data, while the STLF+Random Walk had a robust prediction for the training data. Thus, the SARIMA and STLF+Random Walk models produced a more accurate forecast of LSD outbreaks between 2020 and 2025. From retrospective Space–Time Cluster Analysis of LSD, eight possible clusters were also identified, with five of them located in central part of Ethiopia.

**Conclusion:**

The study’s time series and ST-cluster analysis of LSD outbreak data provide valuable insights into the spatial and temporal dynamics of the disease in Ethiopia. These insights can aid in the development of effective strategies to control and prevent the spread of the disease and holds great potential for improving efforts to combat LSD in the country.

## 1 Introduction

Lumpy skin disease (LSD) is a notifiable viral disease of cattle and buffaloes belonging to the genus Capripoxvirus, family Poxviridae. LSD infection is characterized by skin nodules, pox lesions in the ocular, nasal, and oral mucous membranes and on the surface of internal organs, skin edema, fever, lymphadenitis, and sometimes mortality (1, 2). LSD is caused by a virus which is member of *Capripoxviruses* (CaPVs) having a large double-stranded DNA genomes approximately 151 kb long and contains 156 putative genes. It shares 96–97% genome similarity with its CaPVs counterparts: Sheeppox virus (SPPV) and Goatpox virus (GTPV) (3, 4). The economic impact of LSD is considerable for the livestock industry in affected regions and localities (5, 6). For instance, Molla et al. (7) studied the partial economic impact of the LSD field outbreak in Ethiopia. Their findings indicate that the total economic loss per affected herd was USD 1176, with subsistence farms incurring USD 489 in losses and commercial farms suffering USD 2735 in losses. The results of the study highlight that LSD has a significant impact on the livelihoods of impoverished farmers in Ethiopia.

In the context of epidemiology, it should be noted that LSD is a prevalent endemic disease in Ethiopia (8). The first outbreak of the disease occurred between 1981 and 1983 in the northwestern, western, and central regions of the country (9). Since then, the disease has spread to almost all regions and agroecological zones of the country, as evidenced by outbreak reports (10, 11). LSD is a vector-borne disease transmitted by various vectors, such as flies and ticks. The reoccurrence of the disease is consistently associated with rainfall, the emergence of large numbers of arthropod vectors, and low levels of herd immunity (7, 12, 13). Therefore, it is imperative to conduct a thorough study of the spatial and seasonal (temporal) patterns of LSD outbreaks to gain a comprehensive understanding of the seasonal and geo-transmission dynamics. This knowledge helps for strategic planning for control and prevention the disease. It’s particularly useful in planning LSD vaccination programmes.

Veterinary epidemiology and preventive medicine rely on analyzing disease data that has an implicit spatiotemporal component, such as disease outbreaks, surveillance systems data, and hypothesis-based field research (14, 15). Spatio-temporal analyses of livestock disease can be carried out using, a regression tree analysis model, a space–time permutation (STP) and Poisson spacetime (Poisson ST) models (16–18). A space–time scan statistics have recently become popular for disease cluster detection and evaluation for a wide variety of diseases (19). These models can provide valuable insights into disease dynamics and inform decision-making for risk-targeted control disease programs in livestock populations. Thus, in our study, we utilized the space–time permutation (STP) models from the SaTScan v.10.1 open-source software (19) to scrutinize the spatiotemporal patterns of LSD outbreaks in Ethiopia. This allowed us to identify the specific locations and time periods of the outbreaks, providing a comprehensive understanding of the disease’s spread over the past 13 years. To our knowledge, this study stands as the first to utilize a scan statistics STP model for the identification of spatiotemporal clusters of LSD outbreaks on a national level.

Time series data analysis is an extensive topic in data science that allows us to extract valuable information, make informed forecasting, and comprehend complex patterns concealed in sequential time based data (20–22). Time-series modelling is crucial in the field of epidemiology for predicting disease outbreak dynamics, similar to other fields such as weather forecasting and financial and marketing predictions (20). So far, several time-series models have been used in the prediction of livestock diseases like foot and mouth disease (FMD), canine parvovirus (CPV), LSD and some others (11, 23–25). Autoregressive Integrated Moving Average Models (ARIMA), Seasonal ARIMA (SARIMA), Exponential Smoothing State Space Model (ETS), Seasonal Trend Decomposition procedures based on loess forecasting (STF), Neural Network Autoregression (NNAR), ARMA Errors, Trend, and Seasonality (TBATS), and Hybrid Models are some of the most commonly used methods in time series analysis (23, 26). These models help to understand patterns of timeseries data and make good forecasts.

In the current study, four models were selected to be used for forecasting LSD outbreak incidences; ARIMA, ETS, SARIMA, and STL, based on a strong seasonality evaluation of the data. The ARIMA modelling method is considered one of the most efficient methods for modelling time-series data across different disciplines. ARIMA is a time-series forecasting model that combines autoregressive (AR), moving average (MA), and differencing (I) to capture the trend, seasonality, and random fluctuations in the data. The letters p, d, and q represent the order of autoregression, the degree of difference, and the moving average, respectively. Seasonal ARIMA (SARIMA): an extension of ARIMA that specifically accounts for seasonality in the data (27). The STL model is a robust and versatile technique for decomposing time series and forecasting a decomposed time series, particularly good estimate of the trend and seasonal component that are not distorted by divergent behavior in the data achieved (28, 29). The Exponential Smoothing with Trend and Seasonality (ETS) models are a comprehensive class that encompasses Simple Exponential Smoothing, Holt’s Linear Trend Method, Holt-Winters Method with Additive or Multiplicative Seasonality, and all their damped trend versions. These models consist of a level component, a trend component (T), a seasonal component (S), and an error term (E). The smoothing method calculation applies ETS (Error, Trend, and Seasonality) terms additively or multiplicatively, or omits them entirely (28). So far we found only one study, by Molla et al. (11) employed ETS (A, A, M) (Holt-Winters exponential smoothing) and ARIMA models to forecast 36 months of LSD outbreaks using 16 years of retrospective outbreak data. And they reported that the ARIMA model outperformed ETS (A, A, M). Thus, it is noteworthy to consider the ARIMA model and its seasonal extension, the SARIMA model, and add strong seasonal models like STL and ETS models for comparison.

In general, in the current study, the primary objective of this study was to investigate the spatiotemporal dynamics of LSD in Ethiopia. This was achieved by undertaking a retrospective analysis of outbreak reports spanning the period of 2008 to 2020, as well as conducting a comprehensive geographical cluster analysis of LSD outbreaks using space–time scan statistics. Additionally, the study predicted LSD outbreaks for the period of 2020–2025 using four (ARIMA, SARIMA, ETS, STLF) models.

## 2 Materials and methods

### 2.1 Description of study location

Ethiopia is the largest and most populated country in the Horn of Africa, with a total land cover of 1.1 million square kilometers (km^2^) and an estimated human population of 120.8 million in 2022 (30). It is subdivided into 11 regional states and two chartered cities: the Oromia, Afar, Amhara, Benishangul-Gumuz, Gambella, Harari, Sidama, Somalia, Southwest Ethiopia, Southern Nations, Nationalities and People (SNNP) region, Tigray regions, Addis Ababa (city), and Dire Dawa (city). These regional states are further divided into zonal administration (second-level administration) and districts (the 3rd level administration). By 2022, there were approximately 82 zones and approximately 670 rural and 100 urban districts (31). The districts are further divided into the smallest units of local administration, kebeles and neighborhood associations (peasant associations, “PAs”); there are approximately 15,000 kebeles (5,000 urban dweller associations in towns and 30,000 PAs in rural areas) in the country (2).

In Ethiopia, five noticeable topographic features are the western highlands, western lowlands, eastern highlands, eastern lowlands, and rift valley (2, 16). The topographic features vary from Ethiopia’s roof, Mount Ras-Dejen, with elevations 4,533 m above sea level (m.a.s.l.) to the Denakil depression, which drops as low as 11 m.a.s.l. Ethiopia’s climatic zone is divided into five zones, defined by altitude and temperature: the arid zone covers the desert lowlands below 500 m.a.s.l, the semiarid zone covers areas with an altitude of 500–1,500 m.a.s.l, the semihumid zone covers temperate highlands between 1,500 and 2,500 m.a.s.l, the cool to cold humid zone covers temperate highlands between 2,500 and 3,200 m.a.s.l, and the cold, moist temperate zone covers the Afro-alpine areas on the highest plateaus between 3,200 and 3,500 m.a.s.l (2, 16).

This study covered almost all geographical areas, climatic zones, topographic features, and livestock management systems. Ethiopia has the largest livestock population in Africa, with 65 million cattle (32). Livestock is a key factor in the livelihood of the people of Ethiopia as a major source of animal protein, power for crop cultivation, means of transportation, export commodities, manure for farmland and household energy, and means of wealth accumulation (33). According to a World Bank report (19), the livestock sector contributes up to 20% of the total gross domestic product (GDP), 40% of the agricultural GDP, and nearly 20% of national foreign exchange earnings. There are three predominant livestock management systems: mixed crop-livestock, specialized urban and peri-urban (intensive management), and pastoral/agro-pastoral (extensive management) (33). The mixed crop-livestock farming system is dominant in Ethiopia’s highlands, where a high population of cattle (70–80%) exists, but pastoral/agro-pastoral or extensive management dominates the eastern and western lowlands, and the Rift Valley contributes 20–30% of the cattle population (34).

### 2.2 General description of the outbreak data and analysis

Contextually, the study covers all geographical areas of Ethiopia in the sense that the outbreaks reported were from all regional administrations of Ethiopia. LSD is a notifiable disease that must be reported at the national level to the World Organization for Animal Health (WOAH). The reporting mechanism has two methods: one is directly to the regional laboratories, and the other is through the pyramid of administrative divisions, which means that every PA or Kebeles has Agricultural Development Agents (ADAs) who report the outbreak cases to district veterinary clinics and then the veterinarian in the districts after confirming the cases based on clinical diagnosis and the pathognomonic signs of the diseases, reported to the zonal agricultural office and then to the regional agricultural office, and finally to the Ministry of Agriculture of Ethiopia (MOA), Livestock Department, and Epidemiology Directorate.

Thirteen years (2008–2020) of retrospective data (LSD outbreaks) were obtained from the Epidemiology Directorate of the Livestock Department of the MOA. This outbreak data record contained information on the time of reported, place of reported (region, zone, district), number of outbreaks per year of the reported, number of LSD-positive cases and deaths reported, number of animals at risk, and vaccination status during the outbreak time. It covered a total of 11 regional states, the 2 charted cities, 79 zones and 589 districts.

Outbreak information and livestock data were recorded in Microsoft Excel and analyzed using the R statistical software Base package (v4.3.2) (35). Descriptive analyses, such as the total sum of the outbreaks per year for the last 13 years, the average number of LSD outbreaks per month, and from every district for the 13 years, were analyzed to obtain temporal, seasonal, and spatial information. A comparison was also made between the mean number of LSD outbreaks during two major seasons – after the Ethiopian wet season (September to December, which saw a peak in LSD outbreaks) and dry season (May and April). Additionally, the LSD outbreak incidence in districts was analyzed using 13-years data. The average incidence in a district was calculated by adding all reported outbreaks over the study period and dividing by 13. The LSD outbreak distribution at the district level was mapped over 13 years using QGIS 3.4.4 (36), allowing for better visualization of geographical distribution.

#### 2.2.1 Classical and STL decomposition of time series of LSD outbreaks

The time-series data for the LSD outbreak was analyzed using two different decomposition methods: classical additive time-series decomposition and Seasonal-Trend decomposition using Loess (STL decomposition). The analysis was performed using the ctv package in R programme (37), to determine whether the distribution of LSD outbreaks over time was random or exhibited seasonal cyclic patterns such as seasonality, long-term trend, and irregularity (22). The classical additive timeseries decomposition model was given as: Y_t_ = T_t_ + S_t_ + I_t_; where Y_t_ is the-number of LSD outbreaks at time t, T_t_ is the trend-cycle component at time t, S_t_ is the seasonal component at time t, and I_t_ is the irregular component at time t.

Whereas the Seasonal-Trend decomposition with Loess, where LOESS is LOcal regression, (STL) is a widely used method for decomposing time series data into its seasonal, trend-cycle, and remainder components. The formula Yv = Tv + Sv + Rv represents this decomposition, where Yv denotes the LSD outbreak data (observations), Tv represents the trend variation in the data, and Sv represents the seasonal variation in the data, and Rv represents the remainder component of the data. It utilizes a smoothing method called Loess. The decomposition was further used to create STL forecasting (STLF).

#### 2.2.2 LSD outbreak time series forecasting models

Time series forecasting for LSD outbreaks for 60 months (2020–2025) was estimated using STLF +Random walk, ETS (*x, y, z*), (ARIMA) (p, d, q) and (SARIMA [P, D, Q] [P, D, Q]s) models. The first model used was the nonseasonal ARIMA model with (p, d, q) values estimated by Auto. arima () function, which was selected as the best fit. Accordingly, ARIMA (1, 1, 0) was estimated using Auto. arima () function that was used to forecast the LSD outbreak time series (2008–2020). Second, considering the seasonality of the LSD outbreak time series, we utilized the seasonal ARIMA (SARIMA [P, D, Q] [P, D, Q]s) model. The SARIMA model has two parts (p, d, q), which correspond to the nonseasonal and (P, D, Q) seasonal parts of the model. Then, the auto. Arima () function also predicted the parameters for the seasonal ARIMA model as (SARIMA [3, 0, 0] [2, 1, 0]) [12], which was used to forecast LSD outbreaks (2021–2025).

The ETS model is a powerful tool for analyzing both heterogeneity and non-linearity in time series data. It employs exponentially decaying weighted averages to account for the impact of previous observations on future trends. This model offers a total of 30 possible ETS combinations and generates a complete forecast distribution based on past observations (28). It is written as ETS (*x, y, z*), where: x ∈ {A, M}, y ∈ {N, A, Ad}, z ∈ {N, A, M}; here N stands for “none” (no trend component or no seasonality component), A stands for “additive,” Ad stands for “additive-damped,” and M stands for multiplicative (28). The general approach we followed for forecasting using this model was almost similar to that in ARIMA model. The models were estimated using the ets() function in the “forecast” package (v8.21.1) in the R statistical programme (38). We let the ets() function select the best model which resulted in a fitted model of ETS (A, N, A) and with smoothing parameters α = 0.9999 β = None, and γ = 1e-04. Then LSD outbreaks for five (2021–2025) was forecasted and plotted for visual comparison with the other three models.

The STLF+ Random walk model was used to forecast LSD outbreak data, and it was estimated using the STL() function in the R program forecast package. First, the STLF model decomposed the LSD outbreak time series data and then applied a random walk with drift model to forecast the seasonally adjusted series. The STLF() function was employed with adjusted parameters comprising a t.window of 13 and a s.window set as periodic every 12 months. The model fitting was carried out with robust = true, while forecasting was conducted with (h = 60 months). The output was seasonal “naive” forecasts of the seasonal component, which were automatically generated by the forecast() function. These forecasts were utilized to predict LSD outbreaks for the years 2021–2025.

To evaluate forecast ability of the models, the complete timeseries dataset of LSD outbreaks was partitioned into training and validation datasets. The models were trained on 9 years of empirical data (108 months) to forecast for 2017–2020. Validation data of 48 months (test data) was held out for evaluation. The validation was performed between the predicted values for the years 2017–2020, generated using the training dataset, and the actual test dataset (2017–2020). The comparisons among the predicted and actual values were carried out by plotting line graphs and visual inspection as well as using three evaluation error metrics to verify the accuracy and robustness of the models. And then, the four forecast models’ (ARIMA, SARIMA, ETS, and STLF) performances were compared using four evaluation error metrics such as root mean squared error (RMSE), mean absolute error (MAE), the mean absolute percentage error (MAPE) and mean absolute scaled error (MASE). In forecasting, an error refers to the difference between the actual value and the predicted or estimated value. Two types of scale-dependent errors are used in forecasting: Mean Absolute Error (MAE) and Root Mean Square Error (RMSE). MAE is calculated by finding the average differences between the actual and forecasted values, to avoid negative values RMSE used. In contrast, MASE compares the forecast error with the error of a naive forecast (39). However, we were unable to determine MAPE as our data contained zero counts for some months of the time-series data. It is generally accepted that the lower the error metrics, the better the method (26). The second method for the accuracy test was residual testing (Ljung-Box test). The Box-Ljung test is a statistical test used to diagnose the lack of fit of time series models. It can be defined as: H0:The model does not exhibit lack of fit, Ha: The model exhibits lack of fit. A *p* < 0.05 was considered statistically significant and used to reject the null hypothesis.

#### 2.2.3 Retrospective space–time cluster analysis of LSD

Spatio-temporal analyses of the LSD outbreaks were analyzed using a probability model (space–time permutation model [STP]) using the SaTScanTM (v10.1) software package (40). Using the STP model requires only case data with information about the spatial location and time for each outbreak cases, but does not need information about population at risk, controls or risk factors for the background population like Poisson ST and Bernoulli ST models (19, 40). ST-cluster analysis was performed by uploading the LSD outbreak case file and the location data, or districts’ centroids as an input file. In the meantime, in instances where precise outbreak location coordinates were unavailable, we used the center of the polygon as a substitute. To perform the analysis, we set several parameters. The spatial window, or the area to be scanned, was set to cover a maximum cluster size of 50, 40, 30, 20, and 10% of the population at risk. The scans used districts as the spatial modeling units. The maximum temporal cluster size was set at 50% of the study period (6.5 years), and Scans were conducted for areas of high rates, and 1 year time aggregation unit [adjusted in relation to the shortest district level reoccurrence of LSD outbreak previously recorded in Ethiopia was 1 year (11)].

The ST-scan statistic utilizes a dynamic cylindrical window with a circular geographic base, and the height of the cylinder is proportional to time (40). The observed and expected cases were calculated in the circular window, which is movable across each centroid of districts, assuming they are randomly distributed in space. The clusters were identified by dividing the observed cases in the district by the population at risk as expected cases, with the assumption of no clustering of the null hypothesis. A Monte Carlo simulation (number of replications = 999) of the dataset under the null hypothesis was used to calculate the maximum likelihood ratio function. A value of *p* < 0.05 was considered statistically significant and used to reject the null hypothesis that the stated LSD outbreak cases are randomly distributed in space as inputs. Furthermore, clusters defined by the spatiotemporal models were mapped using the Quantum Geographic Information System (QGIS), an open-source software (41). Geographical data on administrative divisions of Ethiopia were obtained from the Humanitarian Data Exchange (HDX) website (42).

## 3 Results

### 3.1 Geographical and temporal distribution of LSD outbreaks

**Figure 1:**
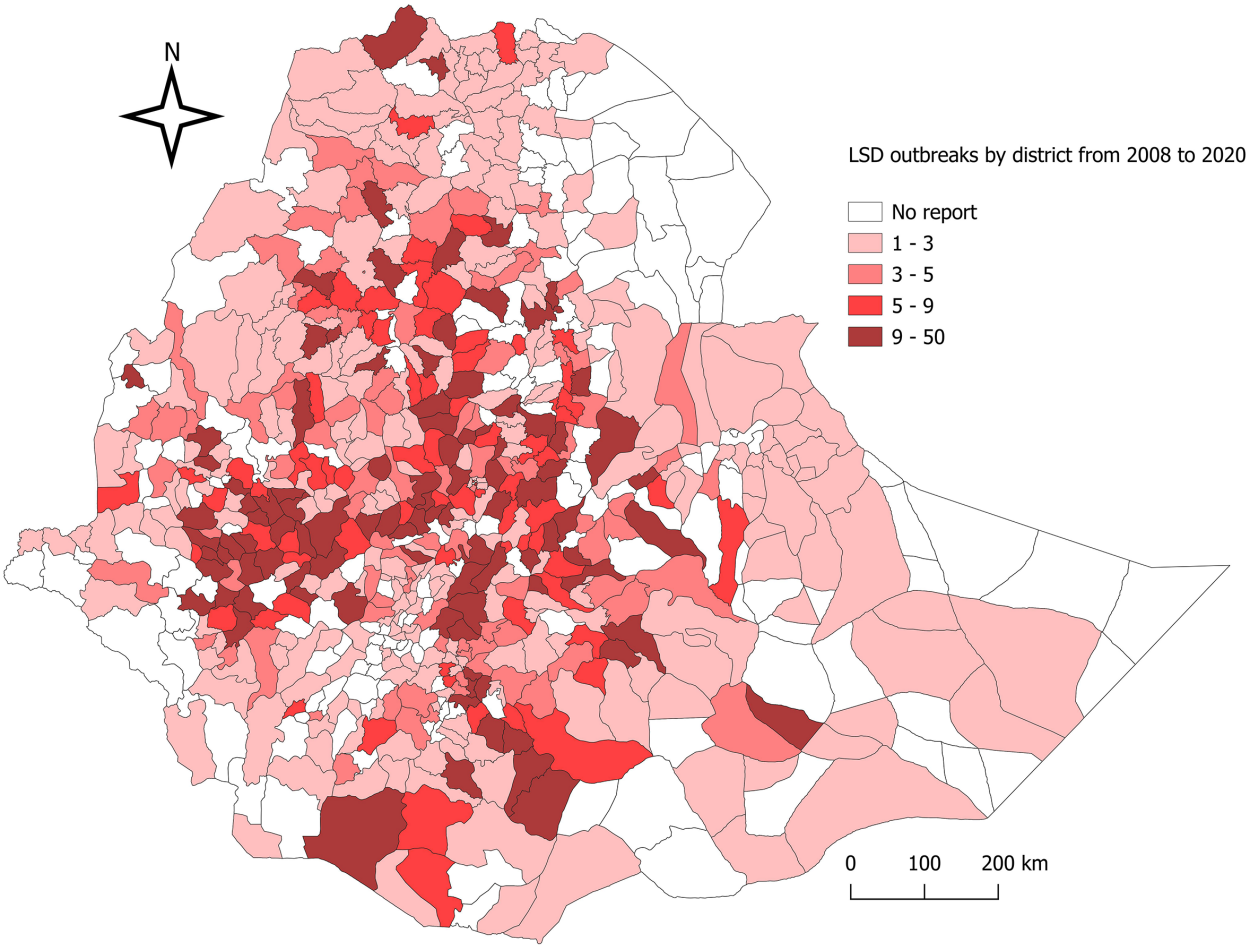
Map of Ethiopia showing the distribution of lumpy skin disease outbreaks, 2008–2020. District-based LSD outbreak distribution per 13 district years in Ethiopia over the 13-year period mapped. The shaded area shows districts with cumulative LSD outbreak reports from 2008 to 2020.

A total of 3,256 LSD outbreaks, 14,754 individual cattle LSD-positive cases, 7,758 deaths, and 289 slaughters were reported over the period 2008–2020 in Ethiopia. The general cattle population at risk was estimated to be approximately 377,17,369 over the period. Although the density of LSD outbreaks might vary among different regions, districts, and PAs, at least one outbreak has been reported from almost all regional states (*n* = 11) and Addis Ababa City in Ethiopia. This covers approximately 68% (527/770) of the districts in the country, indicating a widespread geographical distribution of the disease. At the regional level, Oromia reported the highest number of LSD outbreaks at 57.65% (*n* = 1877), followed by Amhara at 20.64% (*n* = 672), SNNP at 11.73% (*n* = 382), Tigray at 2.73% (*n* = 89), and Somali at 2.52% (*n* = 82). At the zonal level, the North Shewa Zone, Illubabor Zone, and Jimma Zone from the Oromia region ranked in the top three, with 9.52, 9.00, and 5.62%, respectively (Supplementary Table S1). The district-level mapping of LSD outbreaks in Ethiopia between 2008 and 2020 was thorough and effective, as demonstrated in Figure 1. Over the course of 13 years, the average number of outbreaks per district was 6.18, or 0.48 per district per year. Based on Figure 1, the highest incidences were reported in the central part of Ethiopia’s Oromia region, particularly in the Dera district (*n* = 33) in the North Shewa, Becho district (*n* = 26) in the Southwest Shewa, and Chora district (*n* = 23) in the Illubabor Zone of the southwestern part of Ethiopia. The lowest incidences were observed in the eastern lowlands districts of the Afar, Tigray, and Somali regions, as well as the southwest lowlands (Omo Valley).

**Figure 2:**
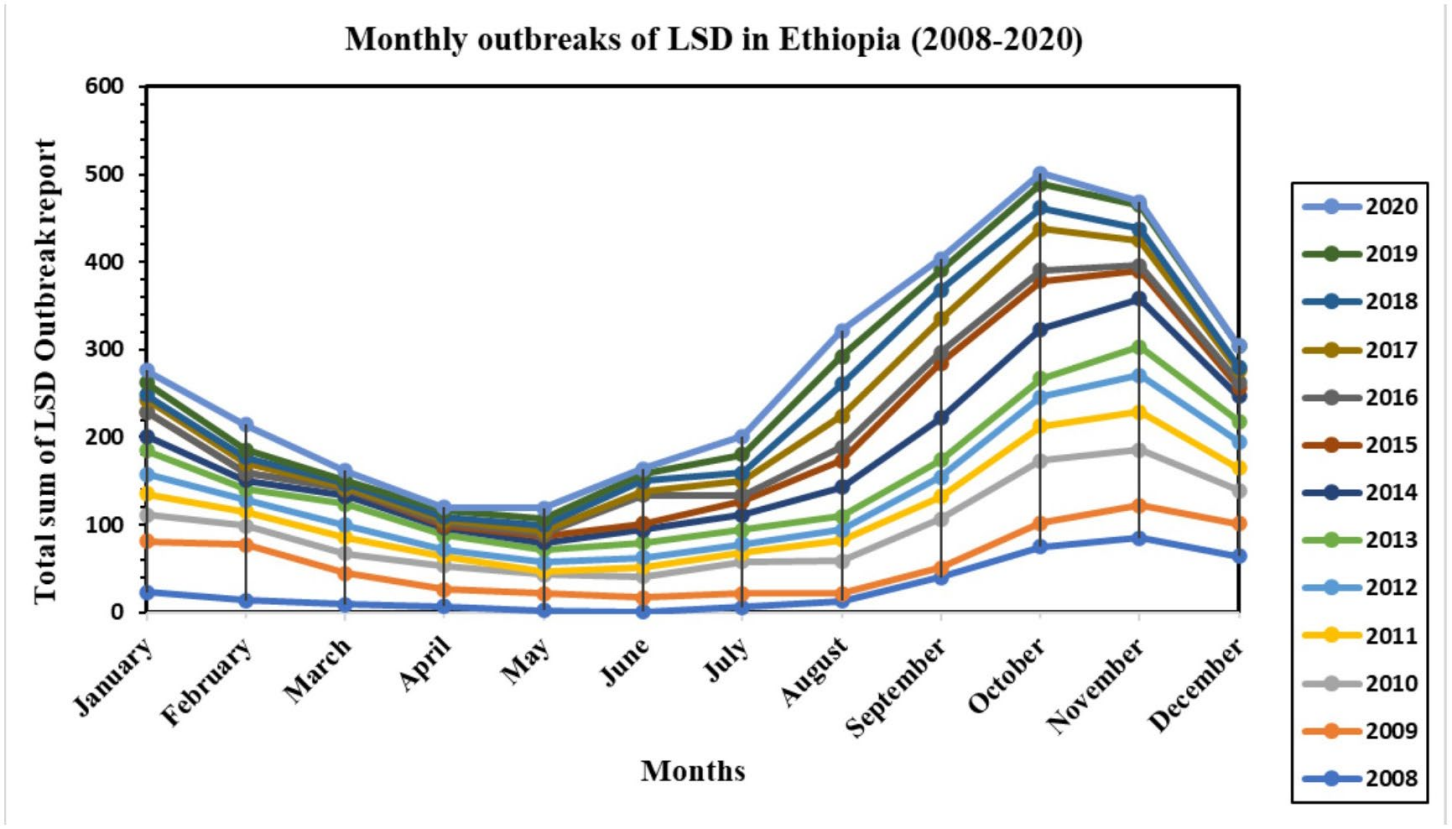
Monthly LSD outbreak distribution over 13 years (2008–2013) in Ethiopia. The line graph shows the monthly seasonality of the LSD outbreak distribution, with the highest recorded in October, November, September, and December (after the rainy season) and the lowest recorded in April and May (dry season). The distribution showed the same patterns for all 13 years of records.

**Figure 3:**
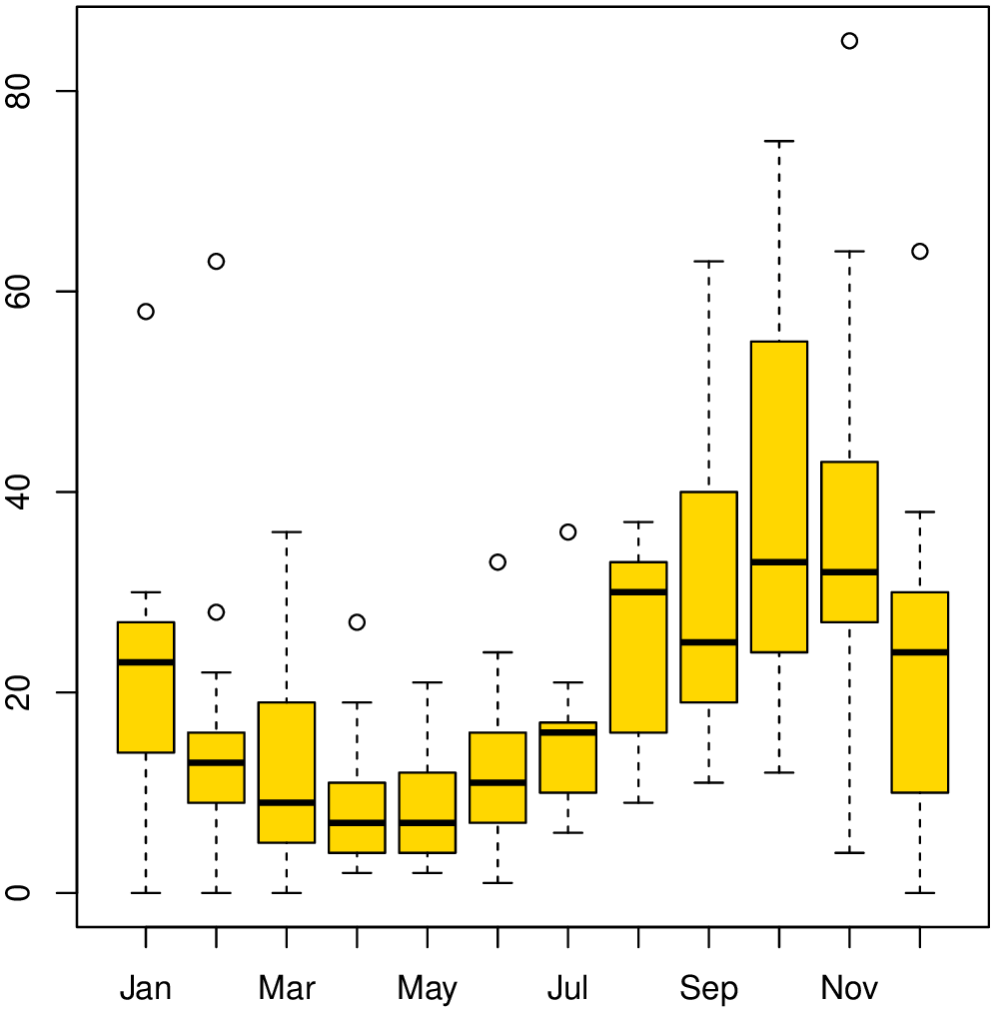
Boxplot for the LSD outbreak time-series data [with windows (width = 800, height = 350)]. Box plots provide a pictorial representation of the seasonal variation in LSD outbreaks in Ethiopia from 2008 to 2020. Each box plot handles intratemporal variation (variation within individual months) and intertemporal variation (between 12 months). Box plots of intertemporal variations indicate how the outbreak distribution varies over various years, whereas intratemporal variations demonstrate the shape of the distribution, its central value, and variability. According to the box plots analyzed in September, October, and November, LSD distributions had very high records of annual variation, and within an individual month, many outbreaks fell above the median values for the same months.

The temporal distribution of LSD disease outbreaks over 13 years, from 2008 to 2020, is presented on a bar graph (see Supplementary Figure S1). The highest number of outbreaks reported per year occurred in 2010 (*n* = 448), while the lowest was in 2016 (approximately 100 outbreak reports). Additionally, a line graph (Figure 2) was used to display the monthly distribution of LSD outbreaks between 2008 and 2020. The purpose was to examine any seasonal or distribution pattern differences among the years. The graph shows a consistent pattern every year, with peak points in October and November and the lowest in April and May. This difference was found to be statistically significant (*p* < 0.05). The outbreak reports increased notably after the rainy season (i.e., September, October, November, and December) and decreased to their lowest level during the dry months of April and May. Additionally, box plots (Figure 3) depict seasonal fluctuations in LSD outbreaks that show intratemporal and intertemporal variations, indicating variation within individual months and between years. The analysis of box plots for September to November revealed high annual variation in LSD distributions, with many outbreaks above median values for the same months.

### 3.2 LSD outbreak time series analysis

#### 3.2.1 Classical and STL decomposition of LSD outbreaks time series data

**Figure 4:**
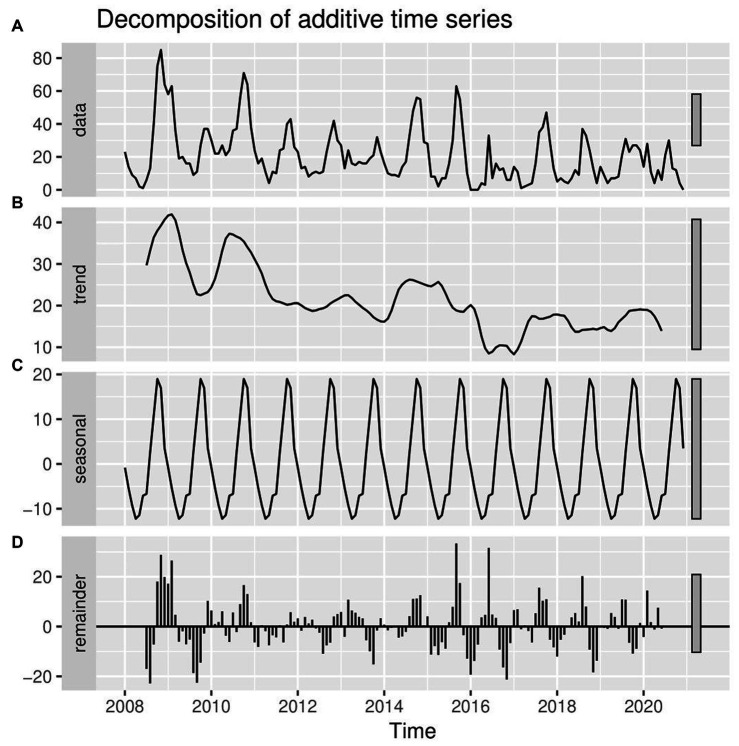
Conventional decomposition of the additive time series of observed LSD outbreaks from 2008 to 2020. **(A)** The graph shows the observed LSD outbreak case numbers (top panel), decomposed into three components (trend, seasonality, and random). **(B)** The 2nd panel (trend) shows a declining pattern from 2008 to 2020. **(C)** The 3rd panel (seasonality) shows strong and regular yearly fluctuations. **(D)** The 4th panel (error panel), or residual components of the time series, is the remainder of the original time series after removing the seasonal and trend time series.

**Figure 5:**
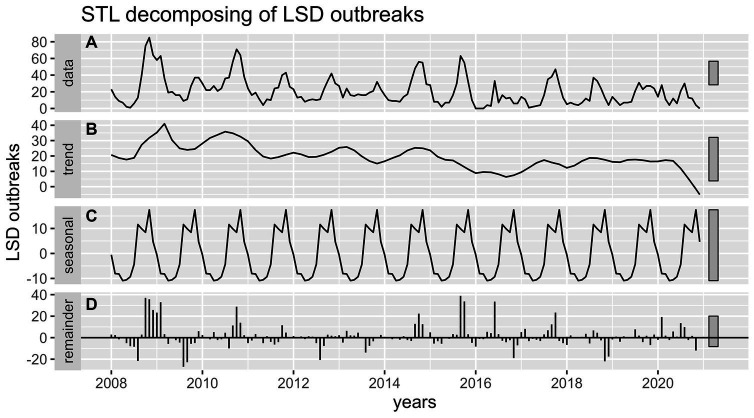
STL decomposition of time series of LSD outbreaks for STL forecasting. **(A)** The graph shows the observed LSD outbreak case numbers (top panel), decomposed into three components (trend, seasonality, and remainder). **(B)** The 2nd panel (trend) shows a weak declining pattern from 2008 to 2020. **(C)** The 3rd panel (seasonality) shows strong and regular yearly fluctuations. **(D)** The 4th panel (error panel), or residual components of the time series, is the remainder of the original time series after removing the seasonal and trend time series.

The most striking finding from the current retrospective study was that LSD outbreaks do not occur at random in time; rather, they have a pattern in time over 13 years. Through both the additive time series decomposition (classical) and STL decomposition of observed LSD outbreaks throughout 2008–2020, the trend, seasonal, and random components were estimated (Figures 4, 5). Based on the visual inspection of Figures 4B, 5B, it is evident that the trend component of the LSD outbreak for the first 4 years (2008–2011) exhibited an observable smooth up-and-down pattern. However, for the next 4 years (2011–2014), the trend remained constant and steady. From 2014 to the end of 2015, there was a slight increase, followed by a decline in 2017. There was a slight increase from the last months of 2017 until the end of the outbreak report (2017–2020), and the trend remained steady. There appears to be a cyclical trend in LSD outbreaks every 2–4 years with an end-to-end decline trend (Figures 4A, 5A). However, both models (classical and STL decomposition methods) showed strong and regular yearly fluctuations of the seasonal component (Figures 4C, 5C) and these fluctuations indicate a seasonal pattern in the epidemics of LSD incidence, which is related to particular seasons (the rainy and dry seasons).

#### 3.2.2 Forecasting of LSD outbreak time series

**Figure 6:**
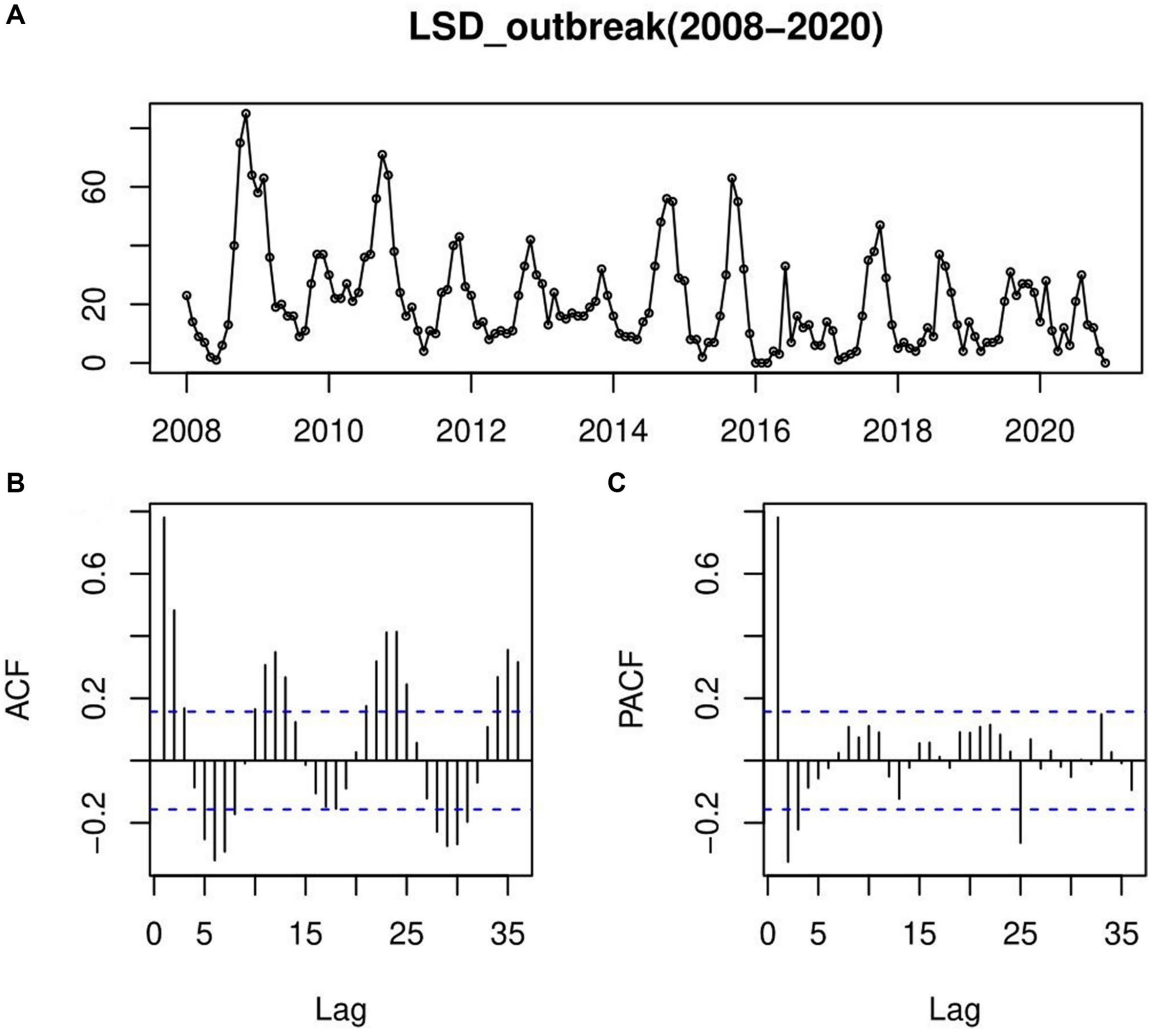
An autocorrelation function (ACF) and a partial autocorrelation function (PACF) plotted to confirm the steady-state prediction of time-series models; **(A)** LSD outbreaks time-series row data plotting, **(B)** autocorrelation function (ACF), the correlogram indicated that there was a significant autocorrelation that decayed slowly over time and rose up again, repeating the pattern like waves indicating pattern of continues in a seasonal form or a regular seasonal cycle that goes from year to year **(C)** partial autocorrelation function (PACF); which has many significant spikes with wave patterns starting from a significant negative PACF value.

In this retrospective LSD outbreak time series finding (Figure 6A), we forecasted the number of LSD outbreaks to occur in each month for the next 5 years (2021–2025). Checking the ACF, the correlogram indicated that there was a significant autocorrelation that decayed slowly over time and rose up again, repeating the pattern like waves. This pattern continues in a seasonal form or a regular seasonal cycle that goes from year to year (Figure 6B). The same was true for the PACF too (Figure 6C).

**Figure 7:**
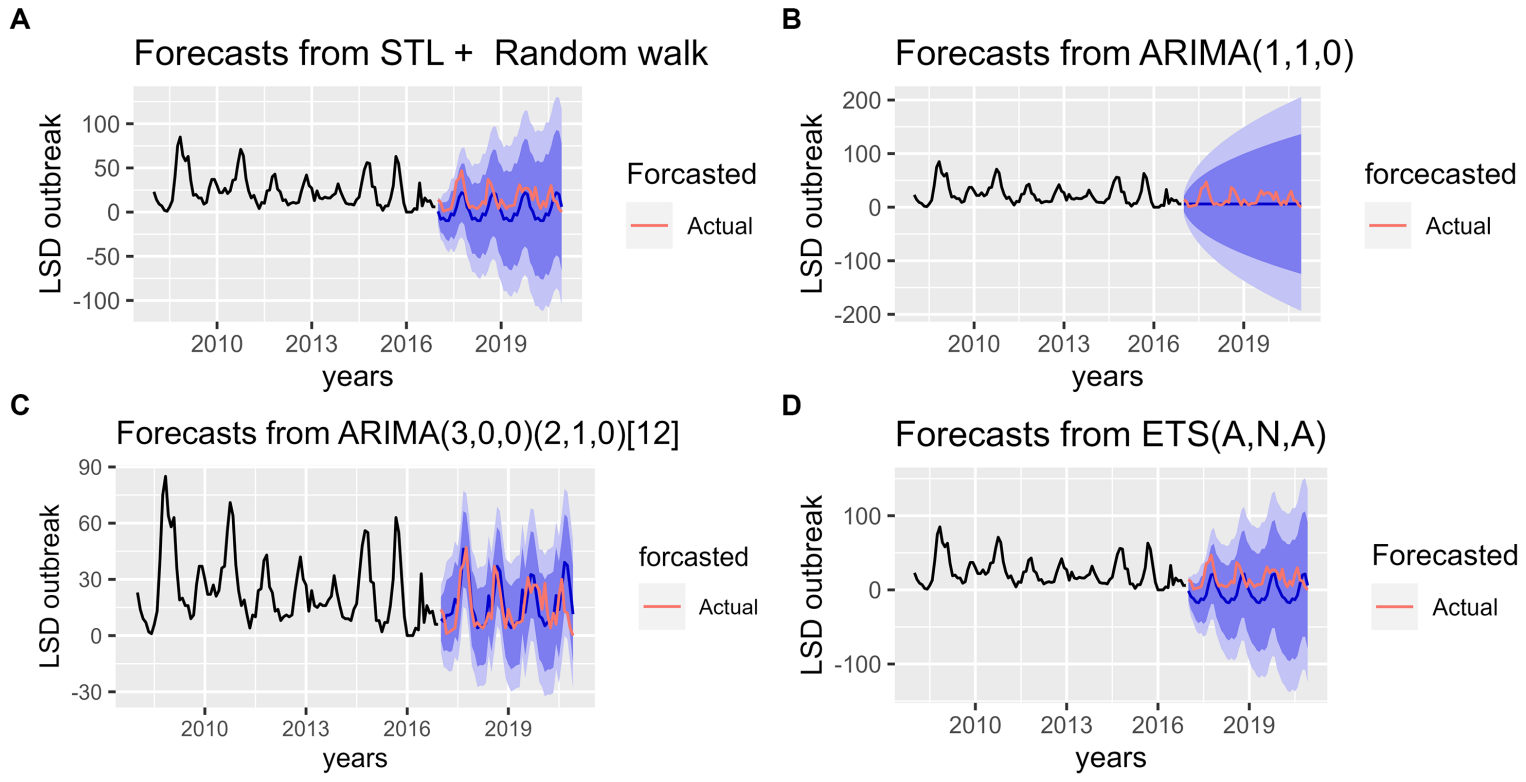
Accuracy tests for the four forecast models for predicting lumpy skin disease outbreak incidence based on the hindcasting method. **(A)** The top left graph shows comparison between forecasted and real data values for STL+ Random walk model. **(B)** The top right graph shows comparison between forecasted and real data values for ARIMA (1,1,0) model. **(C)** The bottom left graph shows comparison between forecasted and real data values for Seasonal ARIMA (3, 0, 0) (2, 1, 0) [12] model. **(D)** The bottom right bottom graph shows comparison between forecasted and real data values for ETS (A, N, A) model. In the graphs the pink line indicates the actual (test) data whereas the blue line indicates the forecasted values.

The training data was fitted, and all four models were plotted with the empirical, forecasted, and validation data shown in Figures 7A–D. The empirical data was displayed in black, forecasted data in dark blue, and validation data in red. Visual inspection of actual and forecast values on the graphs showed that models ETS, STL and SARMA have the same pattern. However, the SARIMA model had a more reliable fit between the forecasted and the actual number of LSD outbreak (2017–2020 years) as its shown in Figure 7C. But for ARIMA model, they have completely different pattern as shown in Figure 7B.

Additionally, the forecasting performance of LSD outbreak time series was evaluated comparatively based on MAE, MASE and RMSEs (Table 1). It was found that using the STL+ random walk method for training data resulted in better performance compared to the other three models. However, the SARIMA model is more precise than other competing models, as demonstrated by the lowest values of the RMSE, MAE, and MASE from the testing data in terms of accuracy. With regarding to residual testing (Ljung-Box test), it was found that except for the ARIMA model other three models (ETS, STLF, SARIMA) models were exhibited fit with Test statistical value *p* > 0.05. This suggests that the three of models are capable of providing a sufficient forecast.

**Table 1:** Error matrices for forecast models accuracy test applied to training and Test LSD outbreak dataset and residual testing (Ljung-Box test).

Forecast models	Training data (2008–2016)	Accuracy test method			Ljung-Box test (residuals test statistics)		
	Test data (2017–2020)	RMSE	MAE	MASE	Q*	*df*	*p*-value
ARIMA (1, 1, 0)	Training set	**11.18**	**8.05**	**0.562**0	46.032	21	0.0013
	Test set	14.76	10.54	0.7360			
SARIMA (3, 0, 0) (2, 1, 0) [12]	Training set	**8.62**	**6.42**	**0.4500**	12.027	17	0.7985
	Test set	10.00	7.71	0.5379			
ETS (A, N, A)	Training set	**8.97**	**6.69**	**0.4671**	18.008	24	0.8026
	Test set	21.90	19.14	1.3367			
STL+ random walk	Training set	**8.14**	**6.00**	**0.4174**	31.434	22	0.0876
	Test set	15.71	13.67	0.9546			

ARIMA, autoregressive integrated moving average; SARIMA, seasonal autoregressive integrated moving average; ETS, error trend seasonality; STL, seasonal-trend decomposition with loess; RMSE, root mean squared error; MAE, mean absolute error; MASE, mean absolute scaled error. The bold values denote the comparison of error matrices, including RMSE, MAE, and MASE, for the training data of LSD outbreaks. Accordingly, STL+ random walk model with lowest value performed best compared to other three models.

**Figure 8:**
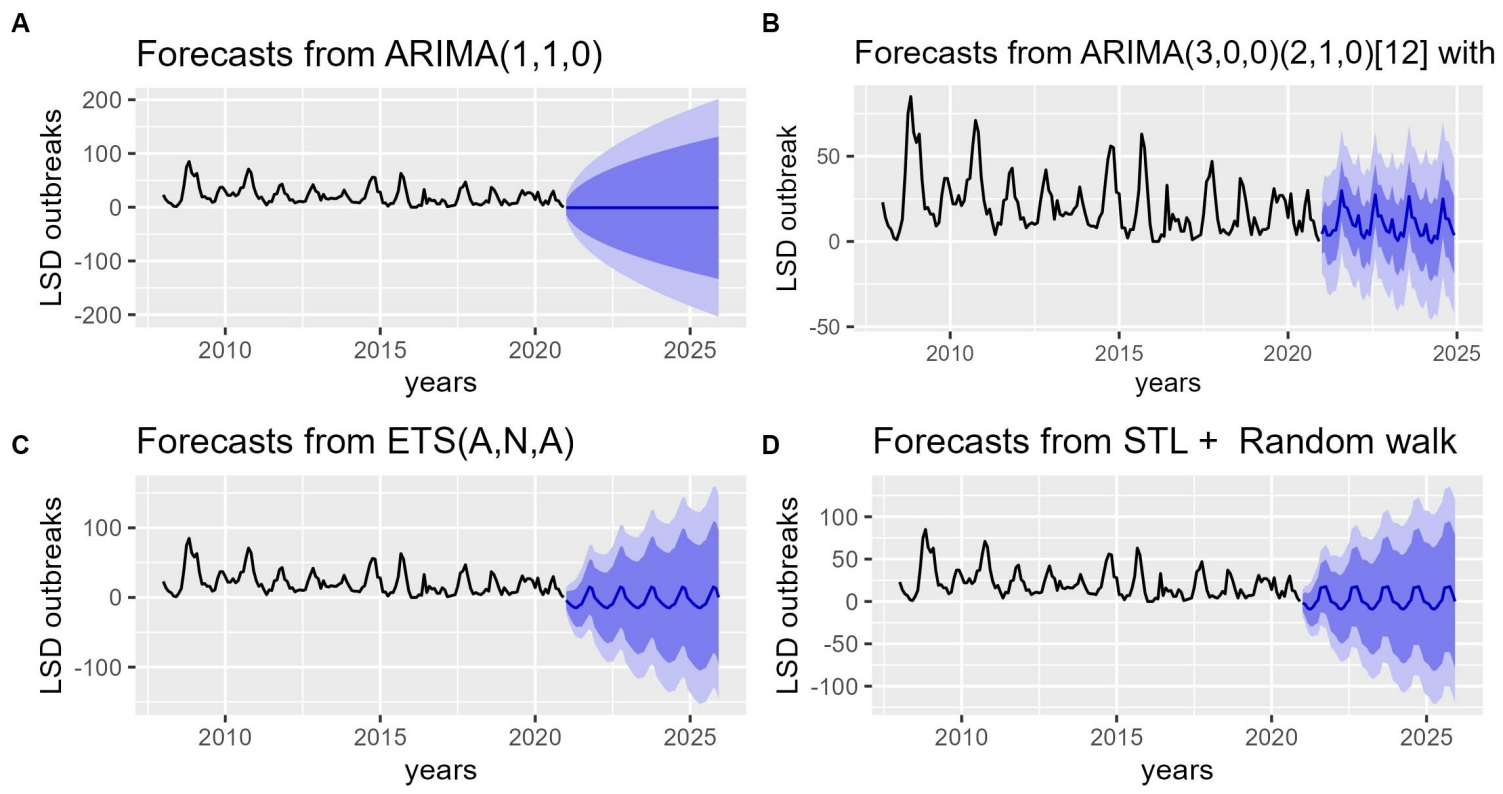
Forecasts of the number of LSD outbreaks (blue lines) from ARIMA, SARIMA, ETS, STL + Random models for the period of 2020–2025. **(A)** The top left graph shows LSD outbreaks forecast from ARIMA (1,1,0) model. **(B)** The top right graph shows LSD outbreaks forecast from seasonal ARIMA (3, 0, 0) (2, 1, 0) [12] model. **(C)** The bottom left graph shows LSD outbreak forecast from ETS (A, N, A) model. **(D)** The bottom right bottom graph shows LSD outbreak forecast for STL+ Random walk model. The plots show future time series prediction for LSD incidence similar to its retrospective data. In the graph the dark blue shaded area represents the 80% confidence interval, while the light blue 95% confidence interval of the predicted values indicates that the trend of LSD, which has exhibited a slight decline, may increase once again to reach its previous peak incidence levels.

After validating the accuracy of all models, predictions for LSD outbreak incidences were made for the years 2021–2025 using all four models (Figure 8). The first model utilized was a nonseasonal ARIMA (1, 1, 0) model. The forecast values were utilized to plot the long-term average (−0.93) of future points in the time series (2021–2025) on a graph (Figure 8A). Thus, visual inspection of the graph appears to be unfavorable, as the result of the forecast is far from desirable. Subsequently, following the estimation of SARIMA parameters, which were determined to be SARIMA (3, 0, 0) (2, 1, 0) [12], the forecast outcomes for LSD outbreaks were plotted, as illustrated in Figure 8B. The forecast graph depicts a more realistic future projection, which exhibits similar fluctuations to those observed in the historical data. The third and fourth forecasts were made using ETS (A, N, A) and STL+ random walk methods. When graphed, the projected values for these forecasts appear similar to those of the SARIMA model, despite some variations in the fluctuation patterns among them as it can be vividly visible in Figures 8B–D.

#### 3.2.3 Retrospective space–time cluster analysis of LSD

In this study, an overall of 804 distinct locations throughout the country and 3,205 LSD outbreak cases, over a period spanning from January 1st, 2008 to December 31st, 2020. Utilizing a spatial window that encompasses a maximum cluster size of 50, 40, 30, 20, and 10% of the population at risk, we have identified eight of the most likely LSD outbreak clusters. These clusters were selected based on their consistency throughout the five spatial scanning window sizes. The results are presented in Figure 8, Table 2 and Supplementary Figures S2–S5.

**Table 2:** Spatio-temporal clusters by retrospective space–time analysis scanning using the space–time permutation model on lumpy skin disease outbreaks in cattle in Ethiopia, 20008-2020.

Cluster 1	Cluster time	Cluster period	# of districts cluster	Centroid (X, Y)/Radius (km)	O	E	O/E ratio	LRT	Value of *p*
Cluster 1	2008/1/1 to 2008/12/31	1 year	75	(9.440026 N, 37.419899 E)/127.61 km	165	55.88	2.95	71.44929	0.0001
Cluster 2	2009/1/1 to 2009/12/31	1 year	14	(7.946830 N, 36.441510 E)/58.57 km	98	24.54	3.99	63.0832	0.0001
Cluster 3	2010/1/1 to 2012/12/31	3 years	70	(7.766095 N, 39.256407 E)/124.58 km	225	101.81	2.21	57.71027	0.0001
Cluster 4	2015/1/1 to 2015/12/31	1 year	4	(9.010225 N, 38.687197 E)/6.02 km	26	3.04	8.54	32.89456	0.0001
Cluster 5	2017/1/1 to 2017/12/31	1 year	26	(9.504936 N, 39.890722 E)/81.72 km	77	10.34	7.44	88.61906	0.0001
Cluster 6	2017/1/1 to 2017/12/31	1 year	133	(14.083459 N, 38.585098 E)/353.87 km	69	22.33	3.09	31.51971	0.0001
Cluster 7	2018/1/1 to 2019/12/31	2 years	45	(3.826794 N, 39.164094 E)/290.38 km	149	25.04	5.95	144.2117	0.0001
Cluster 8	2020/1/1 to 2020/12/31	1 year	26	(7.078917 N, 35.454754 E)/97.21 km	59	5.71	10.34	84.97088	0.0001

The analytical spatial window size was set to cover different cluster sizes (10, 20, 30, 40, and 50%) of the at-risk population, and the temporal scanning covered 50% of the study period. O, observed case; E, expected case; O/E ratio, ratio of observed cases/expected cases; LRT, likelihood ratio test statistics.

Accordingly, the results showed that the most likely primary cluster (Cluster 1) covered (*n* = 75 districts) with in western part of Oromia region and some districts in north-west of Amhara Region within the time frame of January 1st, 2008 to December 31st 2008 with a radius of 127.61 km. The largest most likely cluster on area coverage was cluster 6 (*n* = 133 districts) with radius of 353.87 km, whereas the 2nd was cluster 7 (*n* = 45 districts) with radius of 290.58 km and they are located in the Northern and southern part of Ethiopia crossing into the neighboring countries Eritrea, and Kenya, respectively. The smallest most likely cluster (Cluster 4) has been located in four sub-cities in Addis Ababa (capital city), with a radius of 6.02 km with a time frame from January 1st, 2015 to December 31st, 2015. The long persisted cluster was cluster 3 (*n* = 70 districts) within the time frame of January 1st, 2010 to December 31st 2012 for a duration of 3 years and it coved the central eastern part of Ethiopia, including Arsi and Bale zones of Oromia region. Six of the eight clusters could only persist for a years. Visual analysis in QGIS of 8 LSD outbreak clusters at the district level, where the representative spatial window was the maximum of 30% of the population at risk, was selected and located in Figure 8.

## 4 Discussion

The analysis of spatial data in the present study revealed that the incidence of LSD outbreaks reported in all regions of Ethiopia was consistent with the findings of Molla et al. (11), who reported outbreaks of LSD in all regions of Ethiopia from 2000 to 2015. This finding is significant, as it provides insights into the extent and incidence of the LSD outbreak in Ethiopia, which can aid in the development of effective strategies to control and prevent its spread. Specifically, an endemic form of LSD outbreak in Ethiopia has been predominantly concentrated in three regions, namely, Oromia, Amhara, and the Southern Nations, Nationalities and People’s Region, which account for over 90% of the total LSD outbreak cases. This provides a significant opportunity for targeted intervention efforts aimed at preventing and controlling the spread of the disease. By focusing on these regions, the MOA can maximize the effectiveness of the interventions and minimize the impact of the outbreak at the national level.

When examining the zonal distribution of LSD in the Oromia Regional State alone, it is apparent that the zones most frequently affected by the virus are Illubabor, Jimma, Arsi, and South–West Shewa, as illustrated in Figure 1. These geographic areas are characterized by a midland agro-climate, which is conducive to the breeding of blood-feeding insect vectors that transmit the LSD virus to cattle. It is important to note that the situation has not changed since it was reported by Ayelet et al. (8) and Molla et al. (11). At the district level, the cumulative incidence of LSD outbreaks recorded over a period of 13 years was found to be 6.18, equivalent to 0.48 per district per year. A comparison with the results of Molla et al. (11), who reported an incidence of 5.58 over 16 years or 0.35 per district per year, showed that the incidence recorded in the present study was slightly higher. It should be noted that this finding indicates the persistence of the LSD incidence despite efforts made toward LSD vaccination coverage or the possibility of the current intervention mechanism being ineffective in controlling the disease.

In this study, we examined the temporal distribution pattern of LSD and discovered that despite a general downward trend from 2008 to 2020, the substance exhibited fluctuations among years. However, the decline was statistically significant (*p* = 0.00022). This finding diverges from that of Molla et al. (2), who observed an increasing trend of LSD outbreaks from January 2000 to December 2015. Our results suggest that the LSD outbreak has decreased over time, although it is important to note that fluctuations may still occur year to year. It is possible that the decline might be due to underreported LSD outbreaks from districts and rural kebeles. This may be attributed to political instability in several regional states, particularly after 2017, where the frequency of outbreak reports has significantly decreased. Moreover, vaccination has been the sole LSD control strategy for a long time, and the vaccine type used as a control against LSD has been a live attenuated Kenyan sheep and goat vaccine strain (KS1-180) (43). However, many research findings have reported vaccine failure (11, 43, 44), suggesting that it is difficult to believe that LSD vaccination decreases the incidence of the disease. Therefore, it is noteworthy that despite the overall decrease, the year-to-year variability highlights the need for continued monitoring and intervention efforts.

The seasonal pattern of LSD outbreaks was the second point of consideration. The analysis of LSD outbreaks has revealed a notable seasonal pattern, as evidenced by the classical and STL decomposition methods (Figures 4, 5). Additionally, the data presented in Figures 2, 3 show a consistent pattern of high incidence of LSD following the rainy season annually from 2008 to 2020. Conversely, there was a lower incidence of LSD during the dry season (April and May) consistently during the same time period (11). These outcomes are consistent with Molla et al. (11), which suggests that an increased number of arthropods after the rainy season may be responsible for the rise, while a decrease in the dry season is observed. Many other studies have also proposed that vectors like insects or tick bites transmit LSD (12, 45), which aligns with this finding (12, 45).

In the current study based on our prior understanding of seasonality of LSD incidence from classical and STL decomposition methods, four models (ARIMA, SARIMA, ETS and STLF+Random walk) were utilized for LSD outbreak prediction. According to our finding on predictive models performance evaluation based on four error metrics, STL+ random walk showed better performance for training data, whereas SARIMA model was more precise on validation (Test) LSD outbreak data (Table 1; Figure 7). This suggests that SARIMA appears to be an effective model for short-term forecasting whereas STL model is a robust and flexible method for decomposing and decomposed time series forecasting. This finding is in agreement with previous studies by Punyapornwithaya et al. (23) and Molla et al. (11) who reported SARIMA’s success in short-term forecasting for Foot and mouth disease and LSD, respectively. At the same time outperformance of STL model might be due to division of the forecasting problem into smaller units (46). After validation of the models, the forecasting for LSD outbreak incidences were made for the years 2021–2025 using all four models (Figure 8). Upon visually inspecting Figures 8A–D, it is clear that the SARIMA model provides better predictive values in terms of its lower and upper boundaries as compared to the other three models. However, combined models might be more appropriate for forecasting such disease outbreak incidences to get robust and more accurate outcomes. In the meantime, the capacity of this forecast might be affected by the possibility of LSD outbreaks under reporting, lack of confirmative diagnosis (clinical signed-based case prediction) and some reporting biases on the original data, but it is still very valuable for national vaccination planning in Ethiopia. Upon analyzing the predicted values, it can be inferred that the LSD trend has shown a slight decline. However, it is imperative to note that this trend may escalate again to its previous high peak unless a more strategic control program is implemented in advance. It is crucial to exercise caution and ensure that appropriate measures are taken to curtail the spread of LSD.

**Figure 9:**
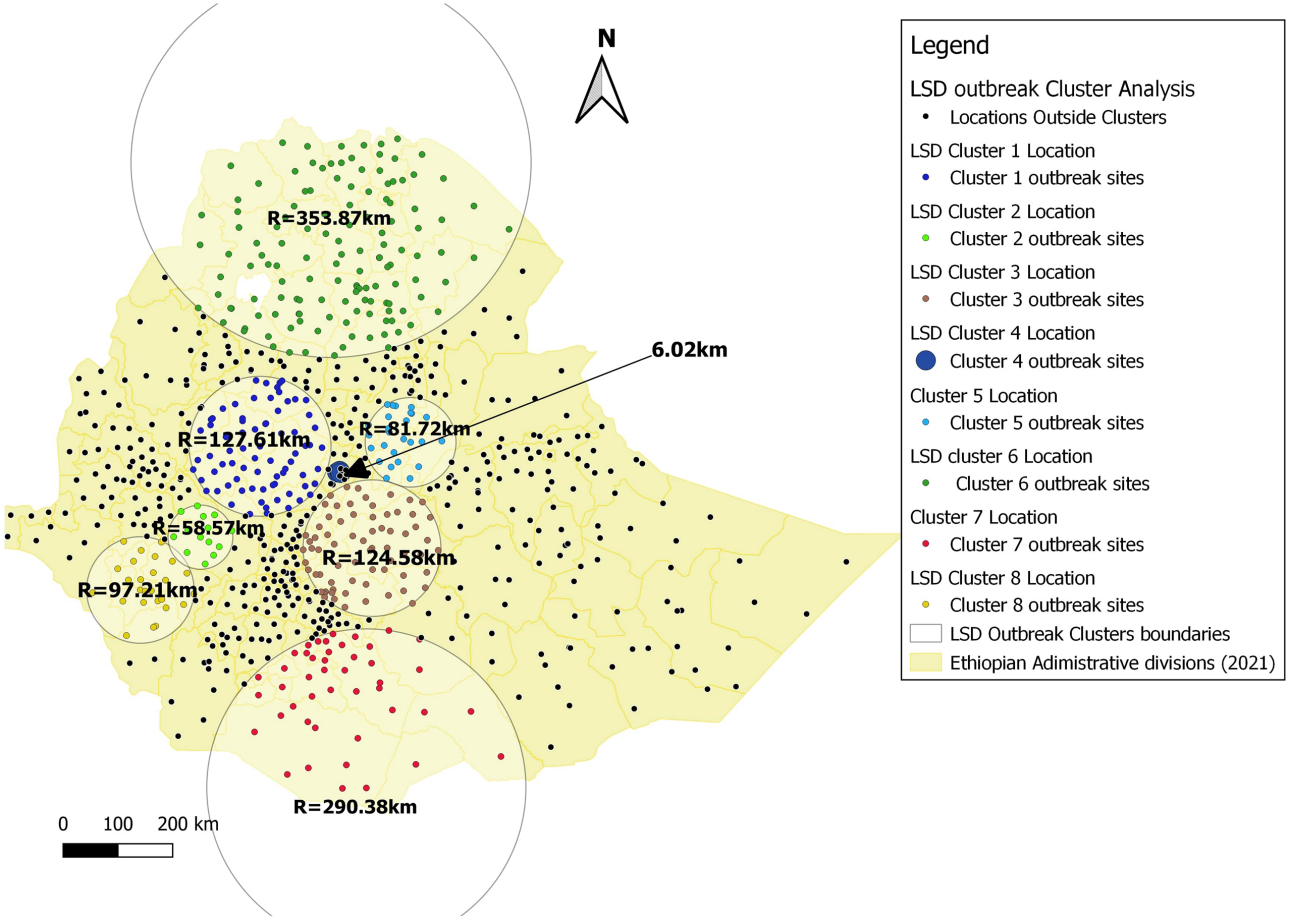
LSD cluster analysis map in Ethiopia (2008–2020). Based on the legends of the map, yellow lines represent the district admirative divisions, whereas the circles indicate the eight SaTScan cluster zones with their radia. The smallest cluster (cluster 4) covered only 6.02 km of radius and with cluster time of 2015/1/1 to 2015/12/31 whereas the largest cluster (Cluster = 6) was with Radius of 358.87 km with cluster time 2017/1/1 to 2017/12/31.

To our knowledge, this study stands as the first to utilize a scan statistics STP model for the identification of spatiotemporal clusters of LSD outbreaks in Ethiopia at national level. However, several studies reported from different regions, including Uganda, Thailand, and the Balkan Peninsula (Greece, Bulgaria, Macedonia, Albania, Serbia and Montenegro), have effectively employed a scan statistics STP model for the analysis of LSD spatiotemporal dynamics (14, 23, 47, 48). In Figure 1, we showed the geographic distribution patterns of LSD outbreaks throughout Ethiopia over 13 years (2008–2020) and we also learned areas with high endemicity of LSD. In this ST-cluster analysis, 8 clusters were identified (Figure 9). The two largest clusters identified on area coverage, namely cluster 6 and cluster 7, are located in the northern part of Ethiopia (Tigray and Amhara regions) and the Southern Ethiopia (Yirga Chefe, Borena and Guji zones). These regions extend towards the borders of neighboring countries such as Eritrea and Kenya, respectively. However, the relatively small number of cases recorded is an indication of the sporadic distribution of LSD occurrence in these clusters. The main challenges in these areas are primarily inhabited by dispersed pastoral communities with underdeveloped infrastructure for intervention in disease control programs (34). Furthermore, areas like the Borena and Guji zones under Cluster 7 are semi-arid and arid environments that are primarily affected by drought, resulting in deficient rainfall and low arthropod population density. The transmission of LSDV might occurred due to animal aggregation around limited water sources and communal grazing areas (rangelands). For instance, Borena plateau (rangeland) one of the best spot of communal grazing (49), which might facilitates the sporadic spread of the LSD disease in this cluster. This statement aligns with the findings of Ochwo et al. (47) who reported that the clustering of animals around limited water sources during dry seasons can act as a facilitator in the transmission of the disease.

In contrast, five clusters representing mixed livestock production systems and warm moist highland, midland and lowland agro-climate zones were identified: cluster 1 (west-central Oromia), cluster 2 (south-west Oromia), cluster 3 (central and south-east Oromia), cluster 4 (central, Addis Ababa), and cluster 5 (north-east Amhara). These regions of the country experience average range humidity (>70%) and average temperature ranging from 8°C to 40°C (50). This creates an environment conducive to the proliferation of different species of arthropods, particularly blood-sucking flies like the common stable fly (*Stomoxys species*). For instance, according to Dawit et al.’s study (52), the density of *S. calcitans* was greater in highland and midland regions, where the average temperature ranged from 8 to 28.5°C. In contrast, *S. ochrosoma* was more abundant in lowland agro-climatic conditions, where the temperature range was 25–40°C. Another laboratory based experimental study conducted by Issimov et al. (51) confirmed that *S. calcitans* required a mean temperature of 26–28°C and humidity levels above 80% to achieve high reproduction rates. This finding highlights the species’ role as the primary vector species for the local transmission of LSDV varying temperatures and humidity in those cluster areas (11).

Regarding temporal patterns, the abundance of Stomoxys flies was higher following the prolonged rainy (wet) season, with a peak density in September and August across most agro-climate zones in Ethiopia, whereas the peak of the LSD outbreak was observed to occur from October to December. Conversely, the population of flies was observed to be markedly low during March, April, and May due to dry and hot climatic conditions, which increase dehydration and are unfavorable for the survival of flies (11). As insects are the primary local transmitters of LSD (12, 13), our findings indicated that the number of LSD outbreaks within indicated clusters has shown influenced by the amount of rainfall, which has an impact on the abundance of flies. Specifically, during periods of high rainfall (June to August), there were fewer LSD outbreaks recorded in those cluster areas. However, following this rainy season (October to December), there was a higher incidence of LSD outbreaks. These outcome is also consistent with Molla et al. (11), as their research indicated an inverse pattern of LSD outbreak when compared to the precipitation pattern, in other words, during the high rainy (wet) season (July and August), the precipitation was high and LSD outbreak was low, whereas from October to December the precipitation was declined but LSD outbreak burden was very high. These results have significant implications for future national vaccination strategies.

Moreover, the cluster 4, located in the central region of Addis Ababa, has the smallest spatial coverage. This cluster may be associated with a high density of commercial farms, particularly dairy and fattening farms (53), which favors mechanical vector transmission of LSD among farms. This finding is consistent with several previous studies that have suggested that the transmission of LSD is likely linked to arthropod vectors that are common in most dairy farms in Central Ethiopia, including arthropod horn flies, *Haematobia irritans* and tick vectors (54). This finding also supported by the study done in Balkan by Mercier et al. (55) that short-distance spread (approximately 7.3 km per week) of LSD virus was associated with cattle movements and presence of a windborne dispersal of virus-carrying vectors (12).

In terms of duration LSD outbreaks in clusters, the longest LSD outbreak persistence recorded in cluster 3 (central and southeast Oromia region) which lasted for 3 years whereas, most of the other clusters persisted only for 1 year. These differences might depend on the variability of LSD local transmission factors including types of cattle breeds, insect vectors burden, effort in disease prevention (vaccination status) and status of uncontrolled animal movement (11, 47). The last cluster (cluster 8) which was located in south-west Ethiopia (SNNP, Kaffa and Sheka Zones) bordering with South Sudan. In this cluster, especially the Kaffa zone (dense forest and high coffee producing area) covered relatively high rainfall which provide wet and humid micro-climates provides suitable environment for multiplication of LSD transmitting flies (56). In general, it has been observed in this study that all the eight clusters identified are mostly located in areas with a high incidence of LSD outbreaks, such as Oromia, Amhara, SNNPS, and Tigray as illustrated in Figures 1, 8. This finding is also supported by previous reports from other researchers (10, 11), who identified a high distribution of the LSD virus in these (10, 11) regions, particularly in specified zones including the central part of Oromia region, Addis Ababa, and the southwest part of Ethiopia, such as Illubabor, Jimma, Aris, and Bale zone. Meanwhile, we have to also consider and treat the issue consciously underreporting might causing missing of some significant clustering (53).

Overall, a retrospective analysis of ST-clusters related to the occurrence of LSD could reveled important insights into the spatiotemporal dynamics of the LSD distribution in time and spaces. Such insights can be useful in developing strategic plans for controlling and preventing LSD in Ethiopia. It is critical to identify and understand the patterns of disease transmission to effectively manage the disease. Therefore, the knowledge generated from this retrospective space–time cluster analysis is an essential tool for disease surveillance and control as well as vector control strategies in Ethiopia. It aided in the identification of high-risk areas in terms of clusters. By using such clusters, we can enhance our interventions including LSD vaccination, vector control and restriction of illegal animal movements and putting strategic surveillance mechanism which ultimately improve the effectiveness of our control strategies.

## 5 Conclusion

The main goal of the current study was to evaluate the spatiotemporal distribution of LSD in Ethiopia based on retrospective outbreak reports from 2008 to 2020. This study aimed to gain a better understanding of the temporal and spatial dynamics of lumpy skin disease based on nationwide LSD outbreak data over the last 13 years (2008–2020). One of the more significant findings to emerge from this study is that throughout its 13 years, the data showed a constant seasonal pattern that was high during and after the rainy season and may be related to arthropod burden. This suggests that, in general, while planning to campaign national LSD vaccination (control), the seasonality of the diseases should be considered. This study also indicated that statistical models such as SARIMA, STLF, and ETS, which were effective for seasonal time-series data, can be used to predict LSD outbreaks in the future. In the meantime, a combined models would give better prediction capacity than single one because each has its own advantages. Additionally, the eight possible clusters identified during space–time cluster analysis of LSD outbreak using STP model can give an insight during designing LSD outbreak intervention at national level. In general, Even though the limitation of this study (underreported outbreaks) might have a denied effect on the whole scope of findings, the findings will benefit livestock authorities in better understanding LSD epidemiology of the disease to enhance efforts and formulate an effective control strategy for preventing future LSD outbreaks.

## Data availability statement

The raw data supporting the conclusions of this article will be made available by the authors, without undue reservation.

## Author contributions

ST: Conceptualization, Data curation, Formal analysis, Investigation, Methodology, Writing – original draft, Writing – review & editing. FR: Conceptualization, Supervision, Writing – review & editing. GB: Data curation, Investigation, Writing – review & editing. SL: Data curation, Formal analysis, Writing – review & editing. JP: Conceptualization, Funding acquisition, Project administration, Supervision, Validation, Writing – review & editing.

## Supplementary material

### Supplementary Figure S1

Annual total sum of LSD outbreaks reported (2008-2020), Ethiopia.

### Supplementary Figure S2

LSD cluster analysis map in Ethiopia (2008–2020). The map shows LSD outbreak clusters, with the spatial window covering a maximum of 10% of the population at risk. In the graph, yellow lines represent the district admirative divisions, whereas the circles indicate the 14 SaTScan cluster zones with their radii.

### Supplementary Figure S3

LSD cluster analysis map in Ethiopia (2008–2020). The map shows LSD outbreak clusters, with the spatial window covering a maximum of 20% of the population at risk. In the graph, the yellow lines represent district administrative divisions, while the circles indicate the eight SaTScan cluster zones with their radii.

### Supplementary Figure S4

LSD cluster analysis map in Ethiopia (2008–2020). The map represents LSD outbreak clusters where representative spatial window was the maximum of 40% of the population at risk. In the graph, yellow lines represent the district admirative divisions, whereas the circles indicate the eight SaTScan cluster zones with their radia.

### Supplementary Figure 5

LSD cluster analysis map in Ethiopia (2008–2020). The map shows LSD outbreak clusters with a spatial window covering up to 50% of the at-risk population. Yellow lines denote district administrative divisions, while circles mark the eight SaTScan cluster zones with their radii.

## References

[1] Radostits OM Gay CC Hinchcliff KW Constable PD. Veterinary Medicine — A Textbook of the Diseases of Cattle, Horses, Sheep, Pigs and Goats, 10th Edition. 10th ed. London: Elsevier Saunders (2007).

[2] Brenner J Bellaiche M Gross E Elad D Oved Z Haimovitz M. Appearance of skin lesions in cattle populations vaccinated against lumpy skin disease: statutory challenge. Vaccine. (2009) 27:1500–3. doi: 10.1016/j.vaccine.2009.01.020 19186204

[3] Tulman ER Afonso CL Lu Z Zsak L Kutish GF Rock DL. Genome of lumpy skin disease virus. J Virol. (2001) 75:7122–30. doi: 10.1128/JVI.75.15.7122-7130.2001, PMID: 11435593 PMC114441

[4] Tulman ER Afonso CL Lu Z Zsak L Sur J-H Sandybaev NT. The genomes of sheeppox and goatpox viruses. J Virol. (2002) 76:6054–61. doi: 10.1128/JVI.76.12.6054-6061.2002, PMID: 12021338 PMC136203

[5] Tuppurainen ESM Venter EH Shisler JL Gari G Mekonnen GA Juleff N. Review: Capripoxvirus diseases: current status and opportunities for control. Transbound Emerg Dis. (2017) 64:729–45. doi: 10.1111/tbed.12444, PMID: 26564428 PMC5434826

[6] Tamire M. Current Status of Lumpy Skin Disease and Its Economic Impacts in Ethiopia. Haramaya University, Haramaya, Ethiopian. Res Vaccine J. (2022). Available at: https://article.scholarena.com/Current-Status-of-Lumpy-Skin.pdf

[7] Molla W de Jong MCM Gari G Frankena K. Economic impact of lumpy skin disease and cost effectiveness of vaccination for the control of outbreaks in Ethiopia. Prev Vet Med. (2017) 147:100–7. doi: 10.1016/j.prevetmed.2017.09.003, PMID: 29254706

[8] Ayelet G Haftu R Jemberie S Belay A Gelaye E Sibhat B. Lumpy skin disease in cattle in Central Ethiopia: outbreak investigation and isolation and molecular detection of the virus. Rev Sci Tech. (2014) 33:877–87. doi: 10.20506/rst.33.3.2325 25812211

[9] Mebratu GY Kassa B Fikre Y Berhanu B. Observation on the outbreak of lumpy skin disease in Ethiopia. Rev Elev Med Vet Pays Trop. (1984) 37:395–9. PMID: 6545834

[10] Gari G Bonnet P Roger F Waret-Szkuta A. Epidemiological aspects and financial impact of lumpy skin disease in Ethiopia. Prev Vet Med. (2011) 102:274–83. doi: 10.1016/j.prevetmed.2011.07.003 21852008

[11] Molla W de Jong MCM Frankena K. Temporal and spatial distribution of lumpy skin disease outbreaks in Ethiopia in the period 2000 to 2015. BMC Vet Res. (2017) 13:310. doi: 10.1186/s12917-017-1247-5, PMID: 29110713 PMC5674741

[12] Sprygin A Pestova Y Wallace DB Tuppurainen E Kononov AV. Transmission of lumpy skin disease virus: a short review. Virus Res. (2019) 269:197637. doi: 10.1016/j.virusres.2019.05.015 31152757

[13] Sprygin AV Fedorova OA Nesterov AA Shumilova IN Byadovskaya OP. The stable fly *Stomoxys calcitrans* L as a potential vector in the spread of lumpy skin disease virus in Russia: short review. E3S Web Conf. (2020) 222:06026. doi: 10.1051/e3sconf/202022206026

[14] Ward MP. Spatio-temporal analysis of infectious disease outbreaks in veterinary medicine: clusters, hotspots and foci. Vet Ital. (2007) 43:559–70. PMID: 20422535

[15] Lian M Warner RD Alexander JL Dixon KR. Using geographic information systems and spatial and space-time scan statistics for a population-based risk analysis of the 2002 equine West Nile epidemic in six contiguous regions of Texas. Int J Health Geogr. (2007) 6:42. doi: 10.1186/1476-072X-6-42 17888159 PMC2098755

[16] Arjkumpa O Suwannaboon M Boonrod M Punyawan I Liangchaisiri S Laobannue P. The First Lumpy Skin Disease Outbreak in Thailand (2021): Epidemiological features and Spatio-temporal analysis. Front Vet Sci. (2022) 8:799065. doi: 10.3389/fvets.2021.799065 35071388 PMC8782428

[17] Nkamwesiga J Korennoy F Lumu P Nsamba P Mwiine FN Roesel K. Spatio-temporal cluster analysis and transmission drivers for Peste des Petits ruminants in Uganda. Transbound Emerg Dis. (2022) 69:e1642–58. doi: 10.1111/tbed.14499, PMID: 35231154

[18] Souley Kouato B Thys E Renault V Abatih E Marichatou H Issa S. Spatio-temporal patterns of foot-and-mouth disease transmission in cattle between 2007 and 2015 and quantitative assessment of the economic impact of the disease in Niger. Transbound Emerg Dis. (2018) 65:1049–66. doi: 10.1111/tbed.12845, PMID: 29508559

[19] Kulldorff M Heffernan R Hartman J Assunção R Mostashari F. A space-time permutation scan statistic for disease outbreak detection. PLoS Med. (2005) 2:e59. doi: 10.1371/journal.pmed.0020059, PMID: 15719066 PMC548793

[20] Brockwell PJ Springer RAD. Introduction to Time Series and Forecasting. 2nd ed. New York, USA: Springer-Verlag New York Inc. (2002).

[21] Rojas Ignacio Pomares Héctor Valenzuela Olga. Advances in Time Series Analysis and Forecasting. 1st ed. Springer (2017).

[22] Kotu V Deshpande B. Time series forecasting In: Data Science. Amsterdam, The Netherlands: Elsevier (2019). 395–445.

[23] Punyapornwithaya V Mishra P Sansamur C Pfeiffer D Arjkumpa O Prakotcheo R. Time-series analysis for the number of foot and mouth disease outbreak episodes in cattle farms in Thailand using data from 2010–2020. Viruses. (2022) 14:1367. doi: 10.3390/v14071367 35891349 PMC9320723

[24] Woldemariyam FT Leta S Assefa Z Tekeba E Gebrewold DS Paeshuyse J. Temporal and spatial patterns and a space–time cluster analysis of foot-and-mouth disease outbreaks in Ethiopia from 2010 to 2019. Viruses. (2022) 14:1558. doi: 10.3390/v14071558 35891538 PMC9322932

[25] Ward MP Iglesias RM Brookes VJ. Autoregressive models applied to time-series data in veterinary science. Front Vet Sci. (2020) 7:604. doi: 10.3389/fvets.2020.00604 33094106 PMC7527444

[26] Perone G. Comparison of ARIMA, ETS, NNAR, TBATS and hybrid models to forecast the second wave of COVID-19 hospitalizations in Italy. Eur J Health Econ. (2022) 23:917–40. doi: 10.1007/s10198-021-01347-4, PMID: 34347175 PMC8332000

[27] He Z Tao H. Epidemiology and ARIMA model of positive-rate of influenza viruses among children in Wuhan, China: a nine-year retrospective study. Int J Infect Dis. (2018) 74:61–70. doi: 10.1016/j.ijid.2018.07.003, PMID: 29990540

[28] Hyndman RJ Athanasopoulos G. Forecasting: Principles and Practice. 2nd ed. Melbourne, Australia: OTexts (2018).

[29] Cleveland RB Cleveland WS McRae JE Terpenning I. STL: a seasonal-trend decomposition procedure based on loess. J Off Stat. (1990) 6:3–33.

[30] United Nations Population Fund. World Population Dashboard Ethiopia. (2022). Available at: https://www.unfpa.org/data/world-population/ET

[31] Districts of Ethiopia. Districts of Ethiopia by 2022. (2022). Available at: https://en.wikipedia.org/wiki/Districts_of_Ethiopia. (Accessed July 26, 2023).

[32] CSA (Central Statistical Authority). Agricultural Sample Survey 2020/21 (2013 E.C). Report on Livestock and Livestock Characteristics. Volume II, Statistical Bulletin 589. Addis Ababa: Scientific Research (2021).

[33] The International Livestock Research Institute (ILRI). Feed the Future Innovation Lab for Livestock Systems Ethiopia’s Livestock Systems Overview and Areas of Inquiry Acknowledgement. Gainesville, FL, USA: Feed the Future Innovation Lab for Livestock Systems. (2021).

[34] Kimball T. Chapter 3 Livestock Production Systems and their Environmental Implications in Ethiopia. Waterville, Maine: Colby College Environmental Studies Program. (2012).

[35] R Core Team. R: A Language and Environment for Statistical Computing. R Foundation for Statistical Computing, Vienna. (2023). Available at: https://www.Rproject.org/

[36] QGIS Developement Team. QGIS Geographic Information System (QGIS 3.32.3 ‘Lima’). Open Source Geospatial Foundation 2009. (2023). Available at: https://www.qgis.org/en/site/forusers/download.html. (Accessed October 27, 2023).

[37] Rob J Hyndman RK. CRAN Task View: Time Series Analysis. Version 2023-12-01. (2023). Available at: https://CRAN.R-project.org/view=TimeSeries

[38] Hyndman R Athanasopoulos G Bergmeir C Caceres G Chhay L O’Hara-Wild M. Forecast: Forecasting Functions for Time Series and Linear Models. R package version 8.21.1. (2023). Available at: https://pkg.robjhyndman.com/forecast/ and https://github.com/robjhyndman/forecast

[39] Hyndman RJ Koehler AB. Another look at measures of forecast accuracy. Int J Forecast. (2006) 22:679–88. doi: 10.1016/j.ijforecast.2006.03.001

[40] Kulldorff M SaTScan TM. User Guide SaTScan User Guide v10.1. (2022). Available at: https://www.satscan.org/

[41] QGIS Development Team. QGIS Geographic Information System. (2023). Available at: https://www.qgis.org/en/site/forusers/download.html

[42] UN Humanitarian Data Exchange Ethiopia - Subnational Administrative Boundaries. (2021). Available at: https://data.amerigeoss.org/dataset/ethiopia-cod-ab. (Accessed January 26, 2024).

[43] Ayelet G Abate Y Sisay T Nigussie H Gelaye E Jemberie S. Lumpy skin disease: preliminary vaccine efficacy assessment and overview on outbreak impact in dairy cattle at Debre Zeit, Central Ethiopia. Antivir Res. (2013) 98:261–5. doi: 10.1016/j.antiviral.2013.02.008, PMID: 23428671

[44] Gari G Waret-Szkuta A Grosbois V Jacquiet P Roger F. Risk factors associated with observed clinical lumpy skin disease in Ethiopia. Epidemiol Infect. (2010) 138:1657–66. doi: 10.1017/S0950268810000506, PMID: 20233495

[45] Chihota CM Rennie LF Kitching RP Mellor PS. Attempted mechanical transmission of lumpy skin disease virus by biting insects. Med Vet Entomol. (2003) 17:294–300. doi: 10.1046/j.1365-2915.2003.00445.x, PMID: 12941014

[46] Theodosiou M. Forecasting monthly and quarterly time series using STL decomposition. Int J Forecast. (2011) 27:1178–95. doi: 10.1016/j.ijforecast.2010.11.002

[47] Ochwo S VanderWaal K Munsey A Ndekezi C Mwebe R Okurut ARA. Spatial and temporal distribution of lumpy skin disease outbreaks in Uganda (2002-2016). BMC Vet Res. (2018) 14:174. doi: 10.1186/s12917-018-1503-3, PMID: 29859091 PMC5984736

[48] Stojmanovski Z. Space-time permutation model applied to the past outbreak data of lumpy skin disease in the Balkan Peninsula from August 2015 to July 2017. Vet Glas. (2018) 72:44–55. doi: 10.2298/VETGL171027003S

[49] Markus H. Rangeland Management in the Borana Pastoral System a Historical Review of Land Management and Development in the Borana zone, Ethiopia. MSc thesis, Wageningen University, the Netherlands. (2013).

[50] Duvallet G Pont A. Stomoxyine flies from Ethiopia. Vet Parasitol. (2008) 153:193–4. doi: 10.1016/j.vetpar.2008.02.010, PMID: 18359569

[51] Dawit L Addis M Gari G. Distribution, seasonality and relative abundance of Stomoxys flies in selected districts of Central Ethiopia. World Appl Sci J. (2012) 19:998–1002. doi: 10.5829/idosi.wasj.2012.19.07.1917

[52] Leta S Mesele F. Spatial analysis of cattle and shoat population in Ethiopia: growth trend, distribution and market access. Springerplus. (2014) 3:310. doi: 10.1186/2193-1801-3-310 25019048 PMC4078045

[53] Kahana-Sutin E Klement E Lensky I Gottlieb Y. High relative abundance of the stable fly *Stomoxys calcitrans* is associated with lumpy skin disease outbreaks in Israeli dairy farms. Med Vet Entomol. (2017) 31:150–60. doi: 10.1111/mve.12217, PMID: 27976815

[54] Mercier A Arsevska E Bournez L Bronner A Calavas D Cauchard J. Spread rate of lumpy skin disease in the Balkans, 2015-2016. Transbound Emerg Dis. (2018) 65:240–3. doi: 10.1111/tbed.12624, PMID: 28239954

[55] Asfaw Y Begna R Masho W. Evaluation of breeding objectives, breeding practices and reproductive performance of indigenous dairy cows in selected districts of Kaffa zone, South West Ethiopia. Vet Med Sci. (2023) 9:2820–34. doi: 10.1002/vms3.1267, PMID: 37728180 PMC10650342

[56] IGAD. Sudan and Ethiopia Draft MoU on Cross-Border Animal Health and Livestock Trade Programmes. Khartoum: Sudan Tiribune (2016).

