# Supplementary material for: Spatiotemporal analysis and forecasting of lumpy skin disease outbreaks in Ethiopia based on retrospective outbreak reports
Source: Front Vet Sci. 2024 Mar 12;11:1277007. doi: 10.3389/fvets.2024.1277007 (PMC10964905; doi:10.3389/fvets.2024.1277007)
Supplement: Supplementary file 1 [file Table_1.DOCX]

**Table 1. LSD outbreak distribution by regions and Zones (2008-2020) in Ethiopia**

| Regions | # of outbreaks over 13 years | % distribution of LSD incidence | Zones | **# of LSD outbreaks at zone level** | **% of LSD outbreaks at zone level** |
| --- | --- | --- | --- | --- | --- |
| Oromia | 1877 | 57.65 | North Shewa | 310 | 9.52 |
| Amhara | 672 | 20.64 | Illubabor Zone | 293 | 9.00 |
| Southern Nations Nationalities and Peoples (SNNP) | 382 | 11.73 | Jimma Zone | 183 | 5.62 |
| Tigray | 89 | 2.73 | West Shewa Oromia | 177 | 5.44 |
| Somali | 82 | 2.52 | Arsi | 165 | 5.07 |
| Benshangul-Gumaz | 50 | 1.54 | Southwest Shewa | 150 | 4.61 |
| Addis Ababa | 43 | 1.32 | Guji Zone | 120 | 3.69 |
| Afar | 38 | 1.17 | South Wollo | 109 | 3.35 |
| Gambela | 15 | 0.46 | East Gojjam | 109 | 3.35 |
| Sidama | 7 | 0.21 | Keffa Zone | 101 | 3.10 |
| Harari | 1 | 0.03 | East Welega | 97 | 2.98 |
|  |  |  | West Welega | 96 | 2.95 |
|  |  |  | East Shewa | 90 | 2.76 |
|  |  |  | Borena | 89 | 2.73 |
|  |  |  | Sidama | 84 | 2.58 |
|  |  |  | South Gondar | 68 | 2.09 |
|  |  |  | West Arsi | 65 | 2.00 |
|  |  |  | Bale | 64 | 1.97 |
|  |  |  | North Gondar | 57 | 1.75 |
|  |  |  | West Gojjam | 57 | 1.75 |
|  |  |  | Agew Awi | 54 | 1.66 |
|  |  |  | West Haraghe | 53 | 1.63 |
|  |  |  | Gurage Zone | 49 | 1.50 |
|  |  |  | Addis Ababa | 43 | 1.32 |
|  |  |  | North West Tigray | 33 | 1.01 |
|  |  |  | Afder (Somali) | 32 | 0.98 |
|  |  |  | East Hararghe | 31 | 0.95 |
|  |  |  | Horo Guduru Welega | 31 | 0.95 |
|  |  |  | Asosa | 31 | 0.95 |
|  |  |  | Oromia special zone in Amhara | 27 | 0.83 |
|  |  |  | Gamo Gofa | 27 | 0.83 |
|  |  |  | Central Tigray | 26 | 0.80 |
|  |  |  | Sheka Zone | 26 | 0.80 |
|  |  |  | Gabi Rasu (Zone 3) | 22 | 0.68 |
|  |  |  | Kellem Wollega | 21 | 0.64 |
|  |  |  | Bench Sheko | 17 | 0.52 |
|  |  |  | Hadiya Zone | 16 | 0.49 |
|  |  |  | Siltie Zone | 11 | 0.34 |
|  |  |  | Wag Hemra | 11 | 0.34 |
|  |  |  | Metekel Zone | 11 | 0.34 |
|  |  |  | Sitti Zone | 11 | 0.34 |
|  |  |  | Nogob Zone | 11 | 0.34 |
|  |  |  | Fafan | 10 | 0.31 |
|  |  |  | Anuak | 9 | 0.28 |
|  |  |  | South Tigray | 9 | 0.28 |
|  |  |  | Hari Rasu (Zone 5) | 8 | 0.25 |
|  |  |  | Kamashi Zone | 8 | 0.25 |
|  |  |  | South Omo | 7 | 0.21 |
|  |  |  | Mekele (special zone) | 7 | 0.21 |
|  |  |  | West Tigray | 7 | 0.21 |
|  |  |  | North Shewa Amhara | 7 | 0.21 |
|  |  |  | East Tigray | 7 | 0.21 |
|  |  |  | Basketo | 6 | 0.18 |
|  |  |  | Korahe Zone | 6 | 0.18 |
|  |  |  | Gedeo | 6 | 0.18 |
|  |  |  | Nuer | 6 | 0.18 |
|  |  |  | Wolayita Zone | 6 | 0.18 |
|  |  |  | Oromia Special Zone Surrounding Finfinne | 6 | 0.18 |
|  |  |  | Dawro | 6 | 0.18 |
|  |  |  | Yem special woreda | 5 | 0.15 |
|  |  |  | Jarar zone | 5 | 0.15 |
|  |  |  | Kembata Zone | 5 | 0,15 |
|  |  |  | Bahir Dar (special zone) | 5 | 0.15 |
|  |  |  | Alaba | 5 | 0.15 |
|  |  |  | Fantí Rasu (Zone 4) | 4 | 0.12 |
|  |  |  | Segen Zone | 4 | 0.12 |
|  |  |  | Argobba special woreda | 4 | 0.12 |
|  |  |  | Dollo Somali | 3 | 0.09 |
|  |  |  | Shabelle Zone | 3 | 0.09 |
|  |  |  | Kilbet Rasu (Zone 2) | 3 | 0.09 |
|  |  |  | Konso Zone | 3 | 0.09 |
|  |  |  | Amaro special woreda | 2 | 0.06 |
|  |  |  | konta special woreda | 2 | 0.06 |
|  |  |  | Awsi Rasu (Zone 1) | 1 | 0.03 |
|  |  |  | Harari | 1 | 0.03 |
|  |  |  | Liben Zone | 1 | 0.03 |
|  |  |  | Burji Zone | 1 | 0.03 |
