# Supplementary figures and images for: Spatiotemporal analysis and forecasting of lumpy skin disease outbreaks in Ethiopia based on retrospective outbreak reports
Source: Front Vet Sci. 2024 Mar 12;11:1277007. doi: 10.3389/fvets.2024.1277007 (PMC10964905; doi:10.3389/fvets.2024.1277007)

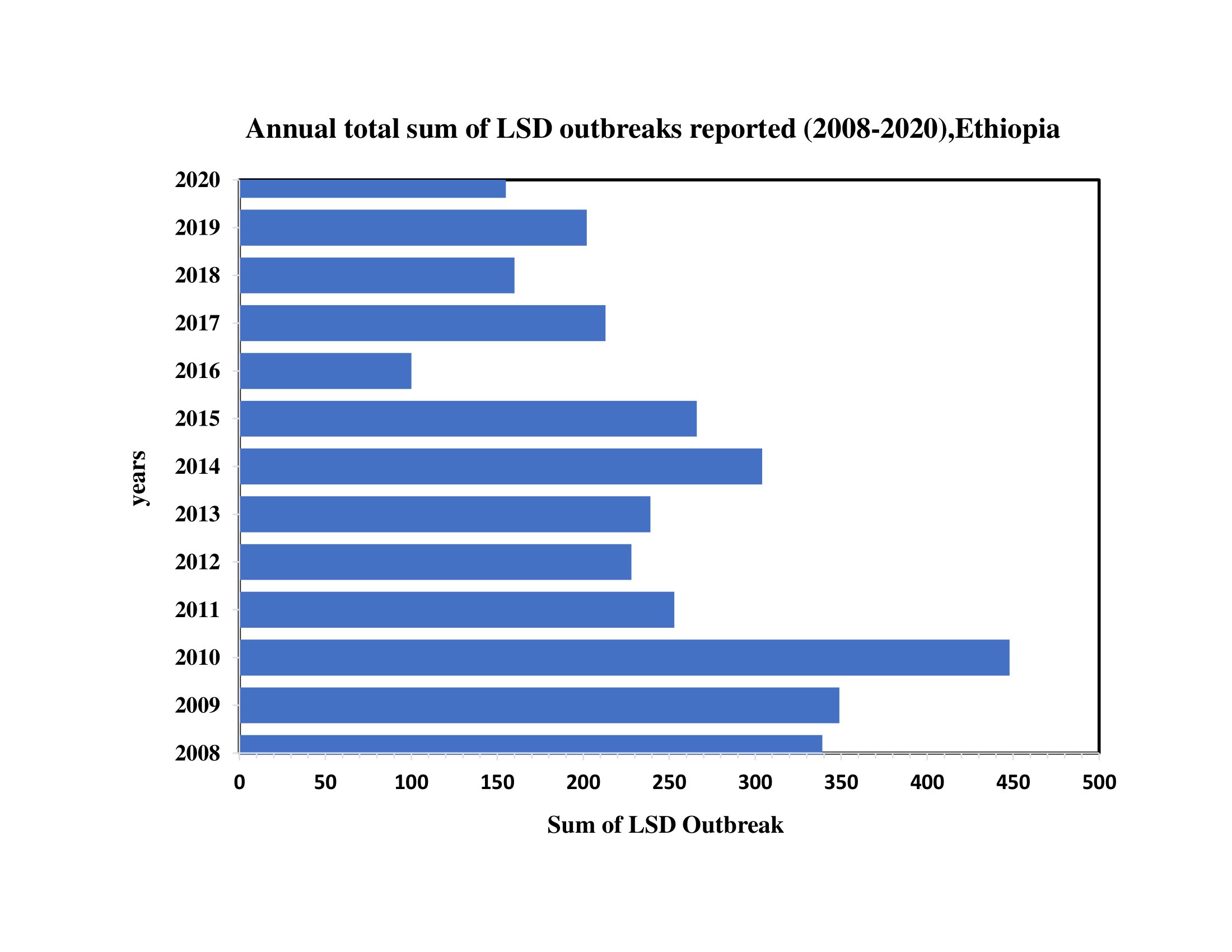

Supplement: Supplementary file 2 [file Image_1.JPEG]

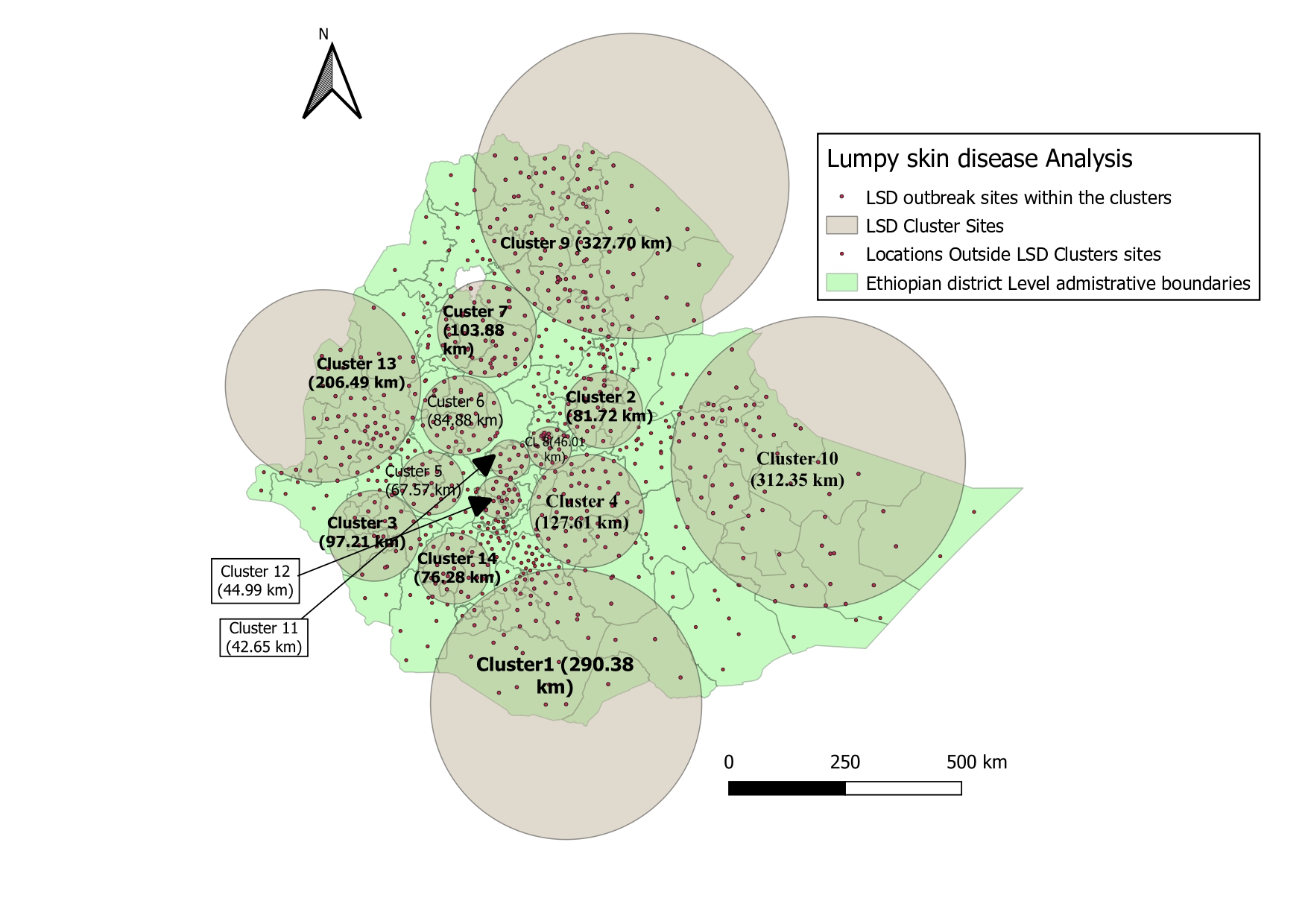

Supplement: Supplementary file 3 [file Image_2.JPEG]

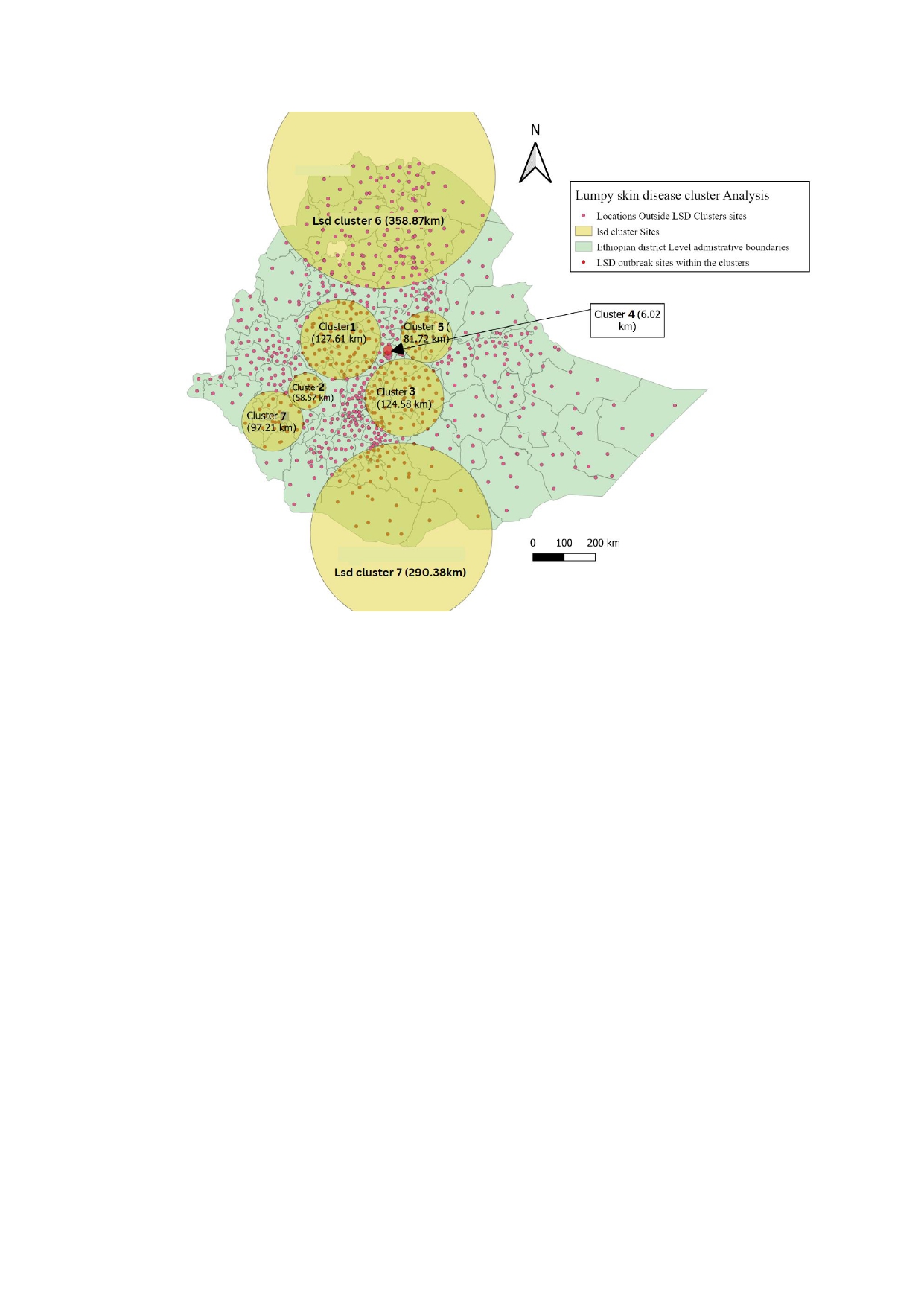

Supplement: Supplementary file 4 [file Image_3.JPEG]
